# A Method for Constructing Nucleosome Arrays with Spatially Defined Histone PTMs and DNA Damage

**DOI:** 10.1002/anie.202500162

**Published:** 2025-04-14

**Authors:** Ziyun Liu, Siqi Xi, Lauren A. McGregor, Kenzo Yamatsugu, Shigehiro A. Kawashima, Jonathan T. Sczepanski, Motomu Kanai

**Affiliations:** ^1^ Graduate School of Pharmaceutical Sciences The University of Tokyo Bunkyo‐ku Tokyo 113‐0033 Japan; ^2^ Department of Chemistry Texas A&M University College Station Texas 77843 USA; ^3^ Department of Biochemistry and Biophysics Texas A&M University College Station Texas 77843 USA; ^4^ Present address: Graduate School of Pharmaceutical Sciences Chiba University, Inohana Chuo‐ku Chiba 260‐8675 Japan

**Keywords:** Abiotic catalysts, Base excision repair, Chromatin, DNA damage, Histone acetylation

## Abstract

DNA damage repair mechanisms, such as base excision repair (BER), safeguard cells against genotoxic agents that cause genetic instability and diseases, including cancer. In eukaryotic nuclei, DNA within nucleosome arrays is less accessible to repair factors than naked DNA owing to the structural constraints of chromatin. Histone acetylation is crucial for loosening the chromatin structure and facilitating access to damaged DNA, yet its effects—particularly in histone globular domains—on BER in nucleosome arrays remain unexplored. Herein, we employ an abiotic/enzymatic hybrid catalyst system (ABEHCS) and a plug‐and‐play strategy to regioselectively introduce histone acetylation and deoxycytidine‐to‐deoxyuridine DNA damage. This approach enables the construction of nucleosome arrays with diverse spatial configurations of histone acetylation and DNA lesions, similar to those found in living organisms. Our findings reveal that H3K56 acetylation in the histone globular domain enhances BER efficiency mediated by UDG and APE1 in nucleosome arrays, contingent upon the spatial relationship between H3K56Ac and the DNA damage site.

## Graphical abstract

This work advances methodology by addressing challenges in mimicking the complex in vivo spatial relationships of histone acetylation and DNA damage. The combination of an abiotic/enzymatic hybrid catalyst system (ABEHCS) and the plug‐and‐play method has constituted a versatile platform to construct nucleosome arrays with precise histone acetylation and DNA damage patterns.

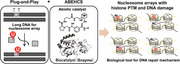

DNA, the carrier of genetic information, is constantly exposed to genotoxic agents, leading to frequent damage. If left unrepaired, this damage causes genetic instability through DNA mutations, potentially resulting in various diseases, including cancer. Among the DNA repair mechanisms, base excision repair (BER) is responsible for identifying and removing a broad range of small lesions caused by oxidation, alkylation, and deamination that modify individual bases with a minimal impact on the DNA double‐helix structure.^[^ ^1^, ^2^ ^]^ BER occurs in a coordinated, stepwise process, beginning with the recognition of chemically modified bases by DNA glycosylases, which cleave the N─glycosidic bond.^[^ ^3^ ^]^ For example, in the case of 2′‐deoxyuridine (dU) generated by cytosine deamination,^[^ ^4^ ^]^ BER proceeds through four main steps^[^ ^5^ ^]^: 1) Uracil DNA glycosylase (UDG) recognizes and removes the damaged nucleotide from DNA, creating an apurinic/apyrimidinic (AP) site; 2) apurinic/apyrimidinic endonuclease 1 (APE1) cleaves the AP site; 3) the repair polymerase Pol β removes the abasic deoxyribose phosphate from the single‐strand break intermediate and replaces the missing nucleotide using the intact strand as a template; and (4) the nicked strand is ligated by DNA ligase I or the DNA ligase III‐XRCC1 complex, completing the repair.

In the eukaryotic nucleus, DNA wraps around histone octamers to form nucleosome structures, with each nucleosome connected by linker DNA to form a nucleosome array. This array then condenses into a higher‐order chromatin structure. Therefore, BER proceeds within the structural constraints of chromatin. Due to the presence of the nucleosome structure and higher‐order chromatin, DNA in chromatin is less accessible to repair factors compared to naked DNA, resulting in inhibited BER at nearly every stage.^[^ ^6^, ^7^, ^8^, ^9^, ^10^ ^]^ Histones undergo various post‐translational modifications (PTMs), such as methylation, ubiquitination, and acetylation, which impact nucleosome structure and chromatin compaction, and thus, the efficiency of BER is supposed to depend on the state of histone PTMs. Notably, histone acetylation has been shown to affect nucleosome stability, mobility, unwrapping, and higher‐order chromatin folding.^[^ ^11^, ^12^, ^13^, ^14^, ^15^ ^]^ This has led to a proposed model in which histone acetylation directly regulates BER by controlling DNA accessibility within chromatin. However, investigating the functional relationship between histone acetylation and BER remains challenging due to the difficulty of selectively manipulating histone modifications at defined regions relative to DNA damage.

One approach to address this issue is to reconstitute nucleosome arrays, which can partly recapitulate physiological chromatin in cells, with both DNA damage and histone modifications and to investigate BER efficiency in vitro. As a technique for site‐specific DNA damage introduction into long DNA of a nucleosome array, we have developed the plug‐and‐play method.^[^ ^16^ ^]^ This versatile DNA manipulation technique involves using a nicking endonuclease to remove a short unmodified DNA fragment and replace it with a synthetic oligonucleotide containing a desired modification, such as dU. To prepare histone proteins with a PTM of interest, various protein engineering approaches, including genetic code expansion, cysteine modification, total chemical synthesis of protein, or protein semi‐synthesis, have been established.^[^ ^17^, ^18^, ^19^ ^]^ Using nucleosome arrays composed of homogeneously acetylated histone H3 (H3K18Ac or H3K27Ac on the histone tail) and a site‐specifically positioned dU, we previously demonstrated that H3K18Ac and H3K27Ac differentially influence the combined activities of UDG and APE1, the initial step of BER.^[^ ^20^ ^]^ However, the role of histone acetylation in the globular domain on BER within nucleosome arrays remains unexplored. Moreover, the canonical reconstitution method is limited to generating nucleosome arrays where all histones carry identical PTMs—a condition unlikely to occur in cells. Considering the diverse spatial positioning of histone PTMs and DNA damage sites within chromatin in vivo, developing nucleosome arrays that replicate such complex spatial relationships is essential (Figure 1a).

**Figure 1:**
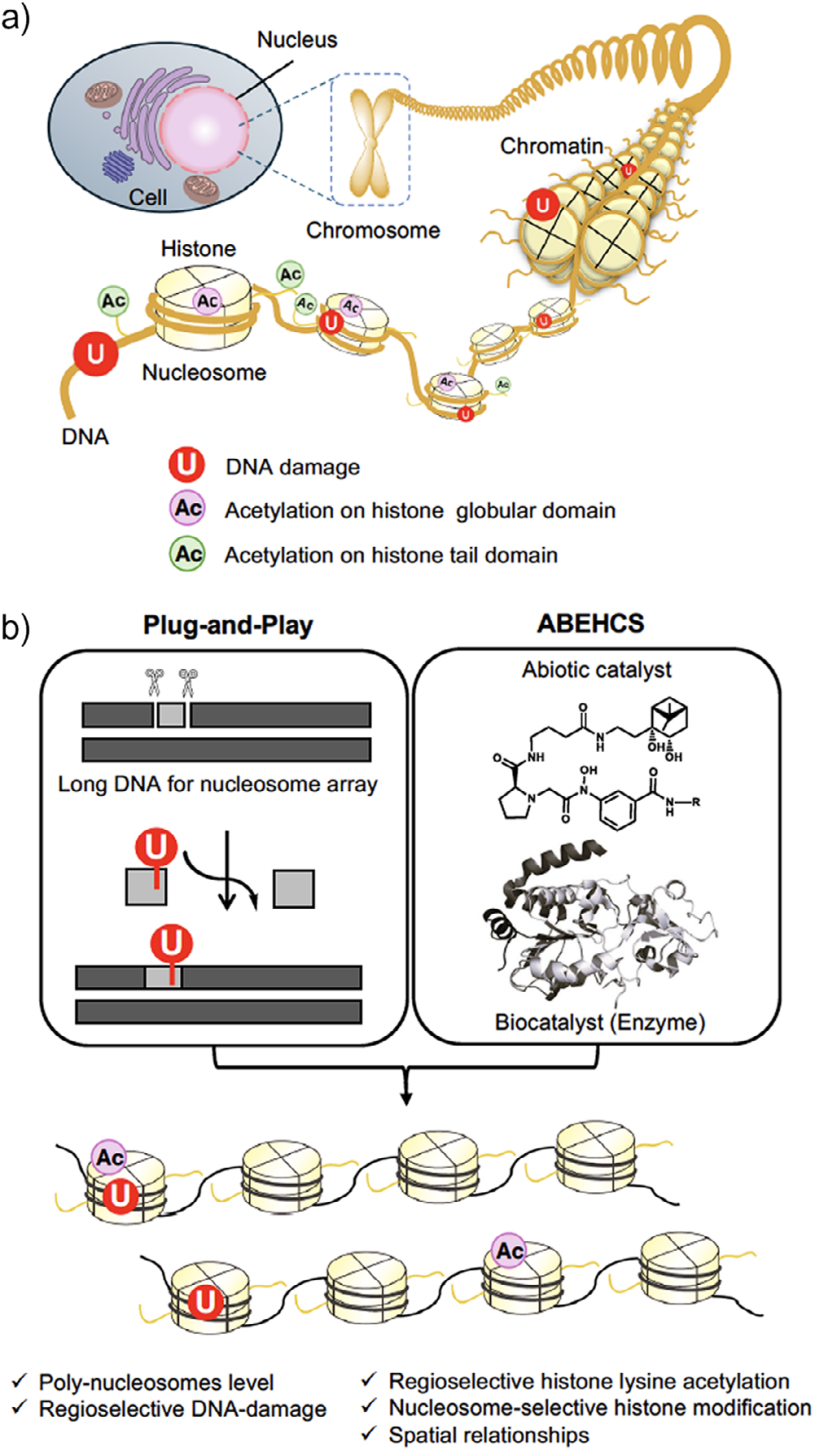
Diverse spatial positionings of histone PTMs and DNA damage sites within chromatin. a) Complex spatial relationship of histone acetylation and DNA damage sites in cells. b) Reconstitution of nucleosome array with variable spatial arrangements of histone acetylation and DNA damage via the combination of an abiotic/enzymatic hybrid catalyst system (ABEHCS) with the plug‐and‐play method.

In this study, we present a chemistry‐based strategy to construct nucleosome arrays with diverse spatial positionings of histone PTMs and DNA damage. This approach combines an abiotic/enzymatic hybrid catalyst system (ABEHCS) with the plug‐and‐play method (Figure 1b). We focused on acetylation at lysine 56 of histone H3 (H3K56Ac), a unique modification implicated in maintaining genomic stability,^[^ ^21^ ^]^ although its specific mechanisms remain unclear. H3K56 is located in the first αN helix of the globular H3 core domain, with its side chain extending toward the DNA major groove near the nucleosome entry and exit sites.^[^ ^22^ ^]^ H3K56Ac weakens the electrostatic interactions between the DNA termini and histones.^[^ ^14^, ^23^, ^24^ ^]^ This modification facilitates spontaneous and transient unwrapping motions of the DNA ends, thereby increasing DNA accessibility.^[^ ^15^, ^24^, ^25^, ^26^, ^27^, ^28^ ^]^ Previous studies have investigated the impact of H3K56Ac on BER using mono‐nucleosomes.^[^ ^29^, ^30^ ^]^ However, its effects on BER in nucleosome arrays remain unexplored. Using our methodology, we examined how H3K56Ac influences BER in nucleosome arrays.

To introduce regioselective histone acetylation in nucleosome arrays, we utilized boronate‐assisted hydroxamic acid (BAHA), an abiotic regioselective lysine acylation catalyst.^[^ ^31^ ^]^ BAHA contains diol and hydroxamic acid moieties, efficiently recruiting and activating chemically stable, boronate‐containing acetyl donors (e.g., **2** in Figure 2a). In the presence of an acetyl donor, BAHA selectively acetylates nearby lysine residues, defined by conjugation to a targeting ligand through a linker motif, with minimal modification to distal, non‐target sites. We hypothesized that a BAHA catalyst conjugated to a pyrrole‐imidazole polyamide (PIP), which binds sequence‐selectively to DNA,^[^ ^32^ ^]^ could achieve regioselective histone acetylation at a lysine residue proximate to the DNA sequence binding to PIP in nucleosome arrays. The sequence‐selective recognition by PIPs is governed by the pairing of pyrrole (Py) and imidazole (Im) residues. The imidazole (Im) residue preferentially binds to guanine (G), whereas the pyrrole (Py) residue interacts with adenine (A), thymine (T), and cytosine (C) without strong discrimination. As a result, the Im/Py pair recognizes G/C base pairs, while the Py/Py pair recognizes A/T or T/A base pairs. To optimize the distance between the BAHA motif and H3K56, we synthesized PIP‐BAHA catalysts containing two types of linkers with different lengths, 2‐aminoethoxyacetyl (AEA): **1** or glycyl (Gly): **3**, between PIP and BAHA motifs (Figures 2a and S1a).

**Figure 2:**
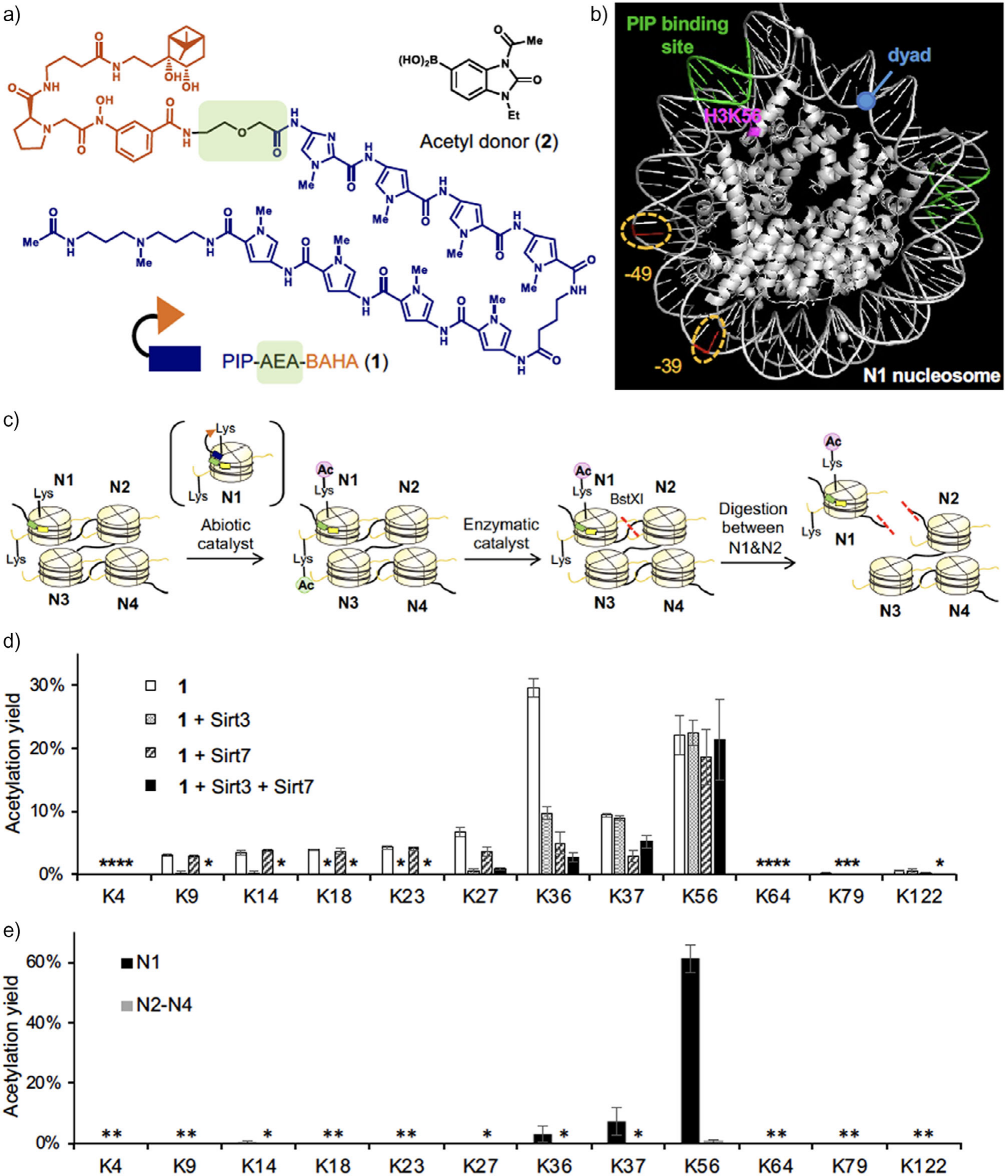
Abiotic/enzymatic hybrid catalyst system (ABEHCS). a) Chemical structures of PIP‐AEA‐BAHA **1** and acetyl donor **2**. AEA: 2‐aminoethoxyacetyl. b) Positions of H3K56 (magenta), PIP‐binding site (green), dU‐39 (red), dU‐49 (red), and dyad (blue) in N1 nucleosome are shown. PIP: pyrrole‐imidazole polyamide. c) Experimental flow. Tetra‐nucleosomes (16 ng µL^−1^ DNA) were treated with PIP‐AEA‐BAHA catalyst **1** (5 µM) and acetyl donor **2** (500 µM) at 37 °C for 16 h (abiotic catalysis). Then, the acetylated nucleosome arrays were treated with Sirt3 (15 ng µL^−1^) and/or Sirt7 (5 ng µL^−1^) at 30 °C for 3 h after abiotic catalysis. For the yield determination at N1 and N2–N4, tetra‐nucleosomes were digested into N1‐nucleosome and N2–N4‐tri‐nucleosomes by BstXI. BAHA: boronate‐assisted hydroxamic acid. d) The average stoichiometry (yield) of each acetylated lysine of H3, analyzed by liquid chromatography‐tandem mass spectrometry (LC‐MS/MS). Error bars represent the standard deviations of three independent experiments. Asterisk (*) indicates not detected. e) The specified stoichiometry (yield) of each acetylated lysine of H3 in N1 and N2–N4, analyzed by LC‐MS/MS. Error bars represent the standard deviations of three independent experiments. Asterisk (*) indicates not detected.

We first optimized regioselective histone acetylation using an undamaged tetra‐nucleosome array composed of four copies of the ‘Widom 601’ nucleosome positioning sequence,^[^ ^33^ ^]^ each separated by 30 bp of linker DNA. A tetra‐nucleosome array is a defined subunit of higher‐order chromatin structures^[^ ^34^ ^]^ and is observed as one of the folding motifs in cells.^[^ ^35^ ^]^ We designed and synthesized a tetra‐nucleosome in which only the 5′‐terminal nucleosome (N1) carried the PIP‐binding DNA sequence (AGTAAT or ATTACT) proximate to H3K56 (Figure 2b, see SI for DNA sequence for the tetra‐nucleosome).

We observed efficient acetylation of H3K56 by PIP‐AEA‐BAHA **1** and acetyl donor **2** (Figure 2c, abiotic catalyst) (Figures 2d and S1b). In comparison, PIP‐Gly‐BAHA **3** produced a lower H3K56 acetylation yield (Figure S1b), suggesting that the Gly linker is less effective than the AEA linker in positioning the BAHA motif near H3K56 when the PIP motif is bound to the target DNA sequence. BAHA conjugated with an unrelated ligand (TMP‐BAHA **4**)^[^ ^31^ ^]^ did not promote H3K56 acetylation, indicating that binding of the catalyst to the nucleosome via the PIP motif is essential for H3K56 acetylation (Figure S1b).

To quantify the acetylation stoichiometry (yield) of histone lysine residues, unreacted lysines in histones purified from acetylated nucleosomes were fully propionylated. The derivatized histones were then digested using trypsin and/or Glu‐C peptidases, and the resulting peptide fragments were analyzed by liquid chromatography‐tandem mass spectrometry (LC‐MS/MS, see Supporting Information for details). Catalyst **1** promoted H3K56Ac with 22.1 ± 3.2% yield, whereas it also promoted acetylation at off‐target lysine residues (K9–K37) within the H3 tail region, with notable yields at K36 (29.6 ± 1.5% yield) and K37 (9.3 ± 0.3% yield) (Figure 2d). This off‐target activity is likely due to the polycationic and highly flexible nature of the histone tail, which binds to DNA through Coulombic interactions. Consequently, lysine residues in the tail may position themselves proximate to the BAHA motif when the catalyst binds to DNA. To mitigate off‐target lysine acetylation, we optimized reaction conditions, including temperature and salt concentration; however, those efforts were unsuccessful.

To address the off‐target acetylation, we explored enzymatic editing of the abiotically acetylated nucleosome array using sirtuin histone deacetylases, particularly Sirt3 and Sirt7 (Figure 2c, enzymatic catalyst). Our previous studies demonstrated that Sirt3 selectively removes lysine acetylation on histone tails, while Sirt7 preferentially deacetylates H3K36 and K37.^[^ ^36^ ^]^ Treatment of the abiotically acetylated nucleosome array with Sirt3 nearly eliminated off‐target acetylation at H3K9–K27; however, approximately 10% acetylation at H3K36 and H3K37 persisted (Figure 2d). Conversely, treatment with Sirt7 significantly reduced acetylation levels at H3K36 and H3K37, but 3%–5% residual acetylation consistently remained across K9–K27 (Figure 2d).

To further improve selectivity, we treated the abiotically acetylated nucleosome array with a mixture of Sirt3 and Sirt7. This combined approach successfully reduced off‐target acetylation at the H3 tail to less than 6%, specifically at H3K36 (Figure 2d). Importantly, the average yield of H3K56Ac remained consistently at ca. 20%, regardless of enzymatic treatment (21.3 ± 6.5% in Sirt3 and Sirt7‐treated sample, Figure 2d). This observation supports our previous finding that H3K56Ac is not a substrate of Sirt7.^[^ ^36^ ^]^ To assess lysine acetylation at other histones, we performed western blotting using an anti‐pan‐acetyl lysine antibody (Figure S1c). The results showed a single acetylated band corresponding to H3, indicating that acetylation of other histones was minimal.

To further analyze the acetylation pattern, the resulting tetra‐nucleosome array was digested with the restriction enzyme BstXI, whose recognition sequence was engineered into the DNA between N1 and N2–N4 (Figure 2c, digestion between N1 and N2). Acetylation yields were quantified using LC‐MS/MS, revealing that H3K56 acetylation proceeded selectively at N1 with approximately 61.3 ± 4.6% yield (Figures 2e and S1d), which corresponds to 15.3% yield by averaging across the tetra‐nucleosomes (vs. 21.3 ± 6.5% observed yield: Figure 2d). Acetylation at N2–N4 was below the detection limit, indicating that the PIP ligand in catalyst **1** selectively directed the BAHA motif to H3K56 in N1. We named this selective acetylation method as an ABEHCS.

To monitor BER activity, we employed the plug‐and‐play method to site‐specifically insert dU labeled with the radioactive isotope P‐32 into DNA and reconstituted nucleosomes.^[^ ^16^ ^]^ Upon treatment with the BER enzymes UDG and APE1, DNA strands were cleaved at dU. We compared BER efficiency across mono‐nucleosome and tetra‐nucleosome arrays containing different relative positions of H3K56 and dU by separating cleaved and uncleaved ssDNA using denaturing PAGE (Figure S2). For dU insertion, we selected two positions: −49, located near the nucleosome entry/exit site (49 nucleotides upstream from the dyad), and −39, positioned farther from the entry/exit site (Figure 2b).

After H3K56 acetylation by the ABEHCS method, we performed ultrafiltration in the presence of 300 mM NaCl before the BER assay. Ultrafiltration removed unbound catalysts, while the addition of 300 mM NaCl might promote the release of nucleosome‐bound catalysts. However, it was challenging to precisely quantify the amount of the remaining catalyst after ultrafiltration. In addition, Sirt3 (∼44 kDa) and Sirt7 (∼45 kDa) were not removed by ultrafiltration due to their molecular size. Therefore, we first confirmed that the remaining catalyst and enzymes did not affect the BER efficiency by comparing BER of tetra‐nucleosomes (dU‐39 at N1) with and without catalyst **1**, Sirt3, and Sirt7 in the absence of acetyl donor **2** (Figure S4). Subsequently, in the following BER assays, we prepared a “Control” sample in which mono‐ or tetra‐nucleosomes were incubated with catalyst **1** in the absence of acetyl donor **2**, followed by incubation with sirtuins. This “Control” sample was then compared with the “Acetylation” sample. The difference in BER efficiency between the “Acetylation” and “Control” samples is solely attributable to H3K56Ac.

In mono‐nucleosomes, a comparison of BER efficiency between dU‐39 and dU‐49 revealed that H3K56Ac enhanced repair at dU‐49 (1.6‐fold) but had no effect on dU‐39 repair (Figure 3a). This result suggests that H3K56Ac specifically facilitates DNA repair at the position near the entry/exit site in mono‐nucleosomes, consistent with the previous research.^[^ ^30^ ^]^ In tetra‐nucleosomes, however, H3K56Ac at the N1 nucleosome promoted the repairs at both dU‐39 and dU‐49 sites within the N1 nucleosome (1.5‐ and 1.5‐folds, respectively: Figure 3b). This observation implies that dU‐39 in the N1 nucleosome is structurally constrained in the tetra‐nucleosome context, and H3K56Ac facilitates BER at this site, likely by inducing structural changes in the tetra‐nucleosome array.

**Figure 3:**
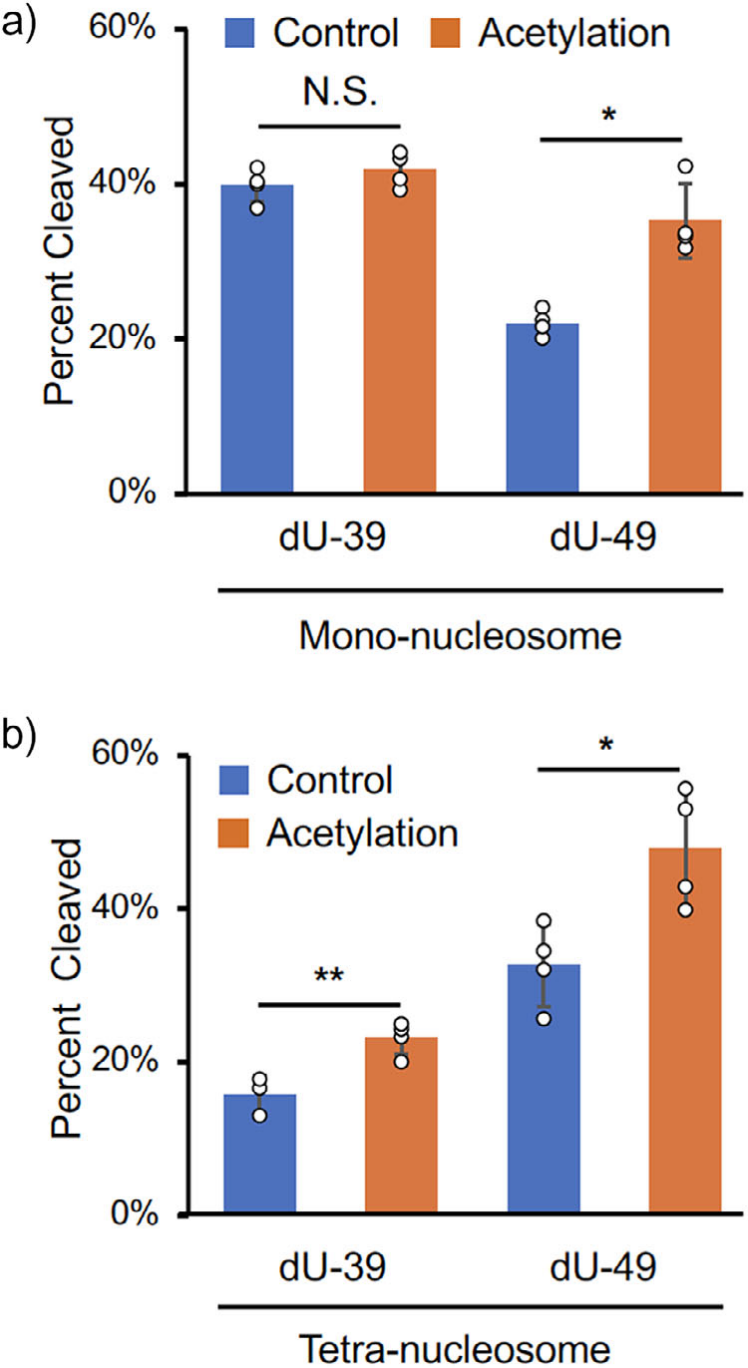
Effects of H3K56Ac at N1 on repair of dU‐39 and dU‐49 by UDG/APE1. a,b) Percentage of dU cleavage by UDG/APE1 in mono‐nucleosomes (a) or tetra‐nucleosomes (b), with or without H3K56Ac. Detailed procedures for BER assay are provided in Figure S2 and the Supporting Information. Each substrate was analyzed in more than three replicates, with data presented as the mean ± standard deviation. Data for samples with H3K56Ac are shown in orange, while those without H3K56Ac are shown in blue. Representative gel images are shown in Figure S8. *p < 0.05, **p < 0.005. N.S., not significant.

Next, we examined BER efficiencies of tetra‐nucleosomes, in which dU‐39 and H3K56Ac were introduced at various spatial positions [Figure 4; dU(1)Ac(1), dU(1)Ac(3), dU(3)Ac(1), and dU(3)Ac(3)]. The efficiencies of BER at internal and terminal nucleosomes in a nucleosome array have not been compared so far. The methods used to construct these tetra‐nucleosomes were analogous to those described in Figure 2c (see Supporting Information for details). Cleavage of dU‐39 at N3 by UDG/APE1 was significantly slower than at dU‐39 at N1 in the absence of histone acetylation (Figure 4, blue bars). This result indicates that dU‐39 at the internal N3 nucleosome is intrinsically much less accessible than dU‐39 at the terminal N1 nucleosome, likely due to structural constraints imposed by the tetra‐nucleosome arrangement. When H3K56Ac was introduced at N3 [dU(3)Ac(3)], the BER efficiency at dU‐39 within the same nucleosome was markedly enhanced (5.0‐fold, Figure 4). This enhancement was substantially greater than the effect observed for dU(1)Ac(1) (1.5‐fold, Figure 4). These findings suggest that H3K56Ac at an internal nucleosome plays a more critical role for UDG and APE1 to access structurally constrained DNA damage sites at an internal nucleosome.

**Figure 4:**
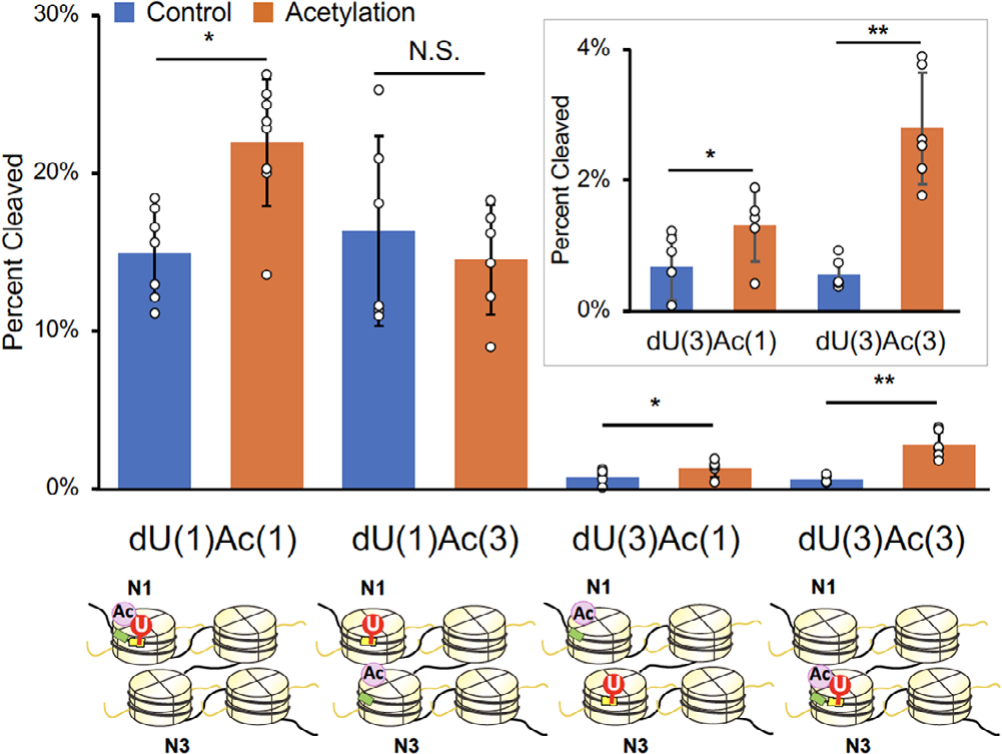
Effects of H3K56Ac on DNA repair by UDG/APE1 in tetra‐nucleosome arrays. Percentage of dU cleavage by UDG/APE1 in four types of tetra‐nucleosomes shown below the horizontal axis. Each substrate was analyzed in more than four replicates, with data presented as the mean ± standard deviation. Data for samples with H3K56Ac are shown in orange, while those without H3K56Ac are shown in blue. The inset contains an enlarged graph of dU(3)Ac(1) and dU(3)Ac(3). Representative data are shown in Figure S8. *p < 0.05, **p < 0.005. N.S., not significant.

We then investigated whether H3K56Ac in one nucleosome could influence BER efficiency in another nucleosome containing DNA damage [dU(1)Ac(3) vs. dU(3)Ac(1) in Figure 4]. Interestingly, H3K56Ac at N1 significantly enhanced BER efficiency at N3 [dU(3)Ac(1): 2.0‐fold], whereas H3K56Ac at N3 had no detectable effect on BER efficiency at N1 [dU(1)Ac(3)] (Figure 4). This finding can be interpreted in light of the observation that DNA damage repair at a less accessible, structurally hindered site (dU‐39 at N3) is more sensitive to the presence of H3K56Ac than repair at a more readily accessible site (dU‐39 at N1). Mg^2^⁺‐dependent sedimentation assay indicated that influences of H3K56Ac in N1 and N3 nucleosomes on the inter‐tetra‐nucleosomal interactions are comparable (Figure S3). Therefore, the differential effects of H3K56Ac between dU(1)Ac(3) and dU(3)Ac(1) are likely due to differences in the local structure within the tetra‐nucleosome rather than differences in the higher‐order structure of tetra‐nucleosome assemblies. Collectively, these results indicate that H3K56Ac promotes BER within nucleosome arrays, with its repair‐enhancing effect strongly dependent on its spatial positioning relative to the DNA damage site. Notably, the effect is more pronounced when the DNA damage resides at a structurally hindered site.

Our findings suggest that H3K56Ac promotes BER through at least two mechanisms. First, H3K56Ac‐mediated enhancement of DNA unwrapping increases the accessibility of UDG and APE1 to DNA damage sites near the DNA entry/exit site.^[^ ^30^ ^]^ Second, H3K56Ac induces local structural changes within the nucleosome array, facilitating access of UDG and APE1 to DNA damage sites even those distant from the DNA entry/exit site, thereby promoting BER. Structural studies of regioselectively acetylated nucleosome arrays would be valuable to further elucidate this mechanism. However, the effects of H3K56Ac on BER in vivo may be more complex, as reader proteins of H3K56Ac, such as the ATP‐dependent chromatin‐remodeling BAF complex,^[^ ^37^ ^]^ may also modulate the accessibility of BER enzymes.

In conclusion, we developed the ABEHCS method to regioselectively introduce histone acetylation into nucleosome arrays. In this study, we utilized two histone deacetylases—Sirt3 for histone tail deacetylation and Sirt7 for H3K36/K37 deacetylation—to remove unintended acetylation by the PIP‐BAHA catalyst, demonstrating the potential of combining abiotic catalysts with enzymes. While we previously investigated the site selectivity of human sirtuins toward lysine residues in the histone H3 and H4 tail regions, their activity toward lysine residues in the histone globular domain remains unexplored. A more comprehensive study of sirtuin site selectivity could further expand their application in regioselective histone acetylation by the ABEHCS method. Combined with the plug‐and‐play method, we constituted tetra‐nucleosome arrays containing histone acetylation and DNA damage at variable relative positions, which partly mimics the scenarios occurring within cells. Using these synthetic tetra‐nucleosome arrays, we investigated the effects of H3K56Ac on DNA damage repair. Our approach allows the use of nucleosome arrays with non‐engineered, wild‐type histones to generate arrays containing diverse combinations of histone acetylation and DNA damage. In principle, this method can be expanded to other types of acylation^[^ ^31^ ^]^ and sites by simply modifying the acyl donor and adjusting the PIP‐binding site within the nucleosome array.^[^ ^38^ ^]^ Combined with the versatility of the plug‐and‐play method, this approach offers broad applicability for producing nucleosome arrays with defined histone and DNA modifications. Further studies are ongoing to broaden the scope of this methodology, enabling its application to a wider range of histone modifications and DNA damage scenarios.

## Supporting Information

The authors have cited additional references within the Supporting Information.^[^ ^39^, ^40^, ^41^, ^42^, ^43^, ^44^ ^]^

## Conflict of Interests

The authors declare no conflict of interest.

## Supporting information



Supporting Information

## Data Availability

The data that support the findings of this study are available in the Supporting Information of this article.
