# Supplementary material for: A Method for Constructing Nucleosome Arrays with Spatially Defined Histone PTMs and DNA Damage
Source: Angew Chem Int Ed Engl. 2025 Apr 14;64(24):e202500162. doi: 10.1002/anie.202500162 (PMC12144863; doi:10.1002/anie.202500162)
Supplement: Supplementary file 1 — Supporting Information [file ANIE-64-e202500162-s001.pdf]

Supporting Information for

A Method for Constructing Nucleosome Arrays with Spatially Defined Histone PTMs and DNA Damage

Ziyun Liu,[1] Siqi Xi,[1] Lauren A. McGregor,[2] Kenzo Yamatsugu,[1†] Shigehiro A. Kawashima,[1]* Jonathan T. Sczepanski,[2,3]* and Motomu Kanai[1]*

[1]Graduate School of Pharmaceutical Sciences, The University of Tokyo; Bunkyo-ku, Tokyo 113-0033, Japan. [2]Department of Chemistry, Texas A&M University, College Station, Texas 77843, United States. [3] Department of Biochemistry and Biophysics, Texas A&M University, College Station, Texas 77843, United States.

correspondence to: skawashima@mol.f.u-tokyo.ac.jp (S.A.K.);

jon.sczepanski@chem.tamu.edu (J.T.S);

kanai@mol.f.u-tokyo.ac.jp (M.K.)

Table of contents

Experimental Section S3
General S3
Materials S3
Synthetic procedures S4
Methods for biochemical experiments S6
Supporting Figures S18
Supporting Tables S27
References S30

Experimental Section

General

Preparative HPLC

Preparative HPLC was conducted by using a JASCO HPLC system equipped with a UV 2075 spectrometer, PU-2086 pumps, a DG-2080-53 degasser, and an MX-2080-32 mixer (JASCO International, Co., Ltd.). Preparative HPLC was performed using YMC-Triart C18 (20 mm I.D. x 250 mm or 10 mm I.D. x 250 mm) columns at 40 °C with a gradient of acetonitrile in 0.1% aqueous TFA, at a flow rate of 10 or 3 mL/min.5% acetonitrile for 5 min, followed by a linear gradient of 5–80% acetonitrile over 75 min. YMC-Triart C18, 230 nm. Retention times (Rt/min.) were recorded.

LC-MS

LC-MS analysis was conducted by using an Agilent Technologies LC-MS (ESI) system equipped with a 1260 Infinity High Performance Degasser, an Agilent 1260 Infinity Binary Pump, a 1260 Infinity Standard Autosampler, a 1290 Infinity Thermostatted Column Compartment, a 1260 Infinity Variable Wavelength Detector, and an Agilent 6120 Single Quadrupole LC-MS (Agilent Technologies, Inc., Santa Clara, CA, U. S. A.).

MALDI-TOFMS

MALDI-TOFMS was obtained with a Shimadzu MALDI-8030.

Synthetic procedures

Solid-phase peptide synthesis (SPPS) of PIP-AEA-Fmoc (S1) and PIP-Gly-Fmoc (S2)

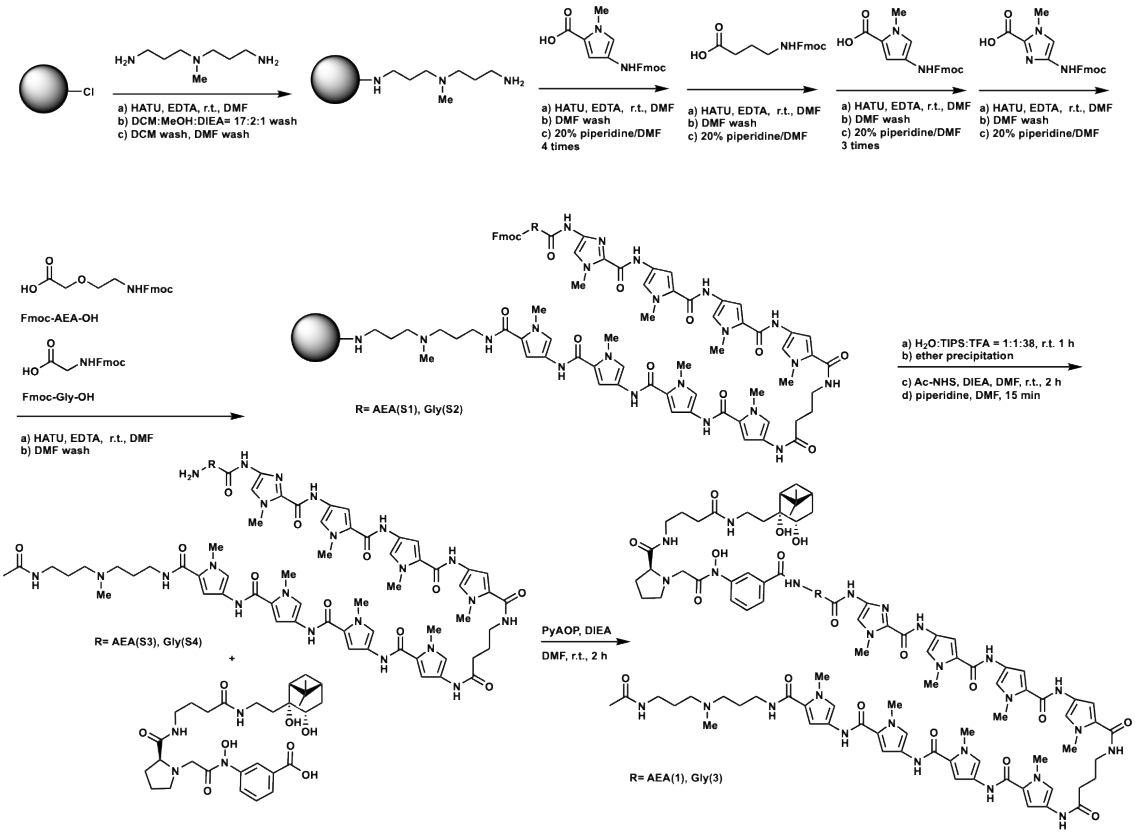

Protected amino acids for the synthesis of PIP were purchased from FUJIFILM Wako Pure Chemical Industries, Ltd. (Osaka, Japan). 2-Chlorotrityl chloride resin was purchased from Peptides International, Inc. (Louisville, KY, USA). To 2-Chlorotrityl chloride resin (139.8 mg, 0.20 mmol, 1.0 eq.) in peptide solid phase synthesis column, DCM (5 mL) was added to and stirred in r.t. for 30 min. 3,3'-diamino-N-methyldipropylamine (300 μL, 1.86 mmol, 9.3 eq.) in DCM (3 mL) was added to the reaction mixture. After being stirred at r.t. for 2 h to overnight, resin was washed by 3 mL DCM:MeOH:DIEA=17:2:1 three times, 3 mL DCM three times, 3 mL DMF three times, 3 mL DCM three times, 3 mL DMF three times. 4-(Fmoc-amino)-1-methyl-1*H*-pyrrole-2-carboxylic acid (Fmoc-Py-OH, 216 mg, 0.6 mmol, 3 eq.), HATU (204 mg, 0.54 mmol, 2.7 eq.) and DIEA (300 μL, 1.72 mmol, 8.6 eq.) in 3 mL DMF were added to reaction column. After being stirred at r.t. for 1 h, Method A was used to cleave Fmoc protection and wash column.

Method A: The reaction column was washed with 3 mL DMF five times, and 2 mL 20% piperidine/DMF was added to the reaction column. After being stirred for 30 s, the solution was discarded, and 2 mL 20% piperide/DMF was added again. After being stirred for 10 min, the solution was discarded again. Then the resin was washed by 3 mL DMF ten times.

The reaction of Fmoc-Py-OH was repeated three times. Then, Fmoc-γ-aminobutyric acid (Fmoc-g-Abu-OH: 195.2 mg, 0.6 mmol 3.0 eq.), HATU (204 mg, 0.54 mmol, 2.7 eq.) and DIEA (300 μL, 1.72 mmol, 8.6 eq.) in 3 mL DMF were added to the reaction column. After being stirred at r.t. overnight, Method A was used to cleave Fmoc protection and column washing. The reaction

of Fmoc-Py-OH was repeated three more times. 4-(Fmoc-amino)-1-methyl-1*H*-imidazole-2-carboxylic acid (Fmoc-Im-OH, 240 mg, 0.66 mmol, 3.3 eq.), HATU (228 mg, 0.6 mmol, 3.0 eq.) and DIEA (592 μL, 3.4 mmol, 17 eq.) in 3 mL DMF were added to the reaction column. After being stirred at r.t. for 2 h, Method A was used to cleave Fmoc protection and wash column. 2-(2-((((9*H*-Fluoren-9-yl)methoxy)carbonyl)amino)ethoxy)acetic acid (Fmoc-AEA-OH, 224 mg, 0.66 mmol, 3.3 eq.) or (((9*H*-Fluoren-9-yl)methoxy)carbonyl)glycine (Fmoc-Gly-OH, 196 mg, 0.66 mmol, 3.3 eq.), HATU(224 mg, 0.6 mmol, 3.0 eq.) and DIEA (592 μL, 3.4 mmol, 17 eq.) in 3 mL DMF were added to reaction column. After being stirred at r.t. for 1 h, Method A was used to cleavage Fmoc protection and wash resin. After being stirred at r.t. for 1 h, the reaction mixture was washed by 3 mL DMF five times. The resin with uncleaved peptide can be stored at -30°C after being washed by MeOH three times. The resin was dried by a vacuum pump. A solution of 125 μL MilliQ water, 125 μL TIPS and 4.75 mL TFA was added to the dried resin and reacted at r.t. for 1 h to cleave the peptide from the resin. Then, the reaction mixture was filtered and evaporated under reduced pressure. 30 mL diethyl ether was added to the residue, and crude **S1** (176.5 mg, 0.100 mmol, 50.0 %) or **S2** (224.2 mg, 0.131 mmol, 65.5 %) as pale brown precipitate was collected. The crude material was used in the next step without further purification.

<u>Synthesis of PIP-AEA-BAHA (1) and PIP-Gly-BAHA (3)</u>

To a stirred solution of obtained crude **S1** or **S2** in 5.00 or 6.53 mL DMF, succinimidyl acetate (157.5 mg, 1.0 mmol, 10 eq.; 206.0 mg,1.31 mmol, 10 eq.) and DIEA (87.1 μL, 0.50 mmol, 5.0 eq.; 114.0 μL, 0.655 mmol, 5.0 eq.) were added and the mixture was stirred at r.t. for 4 h. Then, 1.00 mL or 1.31 mL piperidine was added to the reaction mixture. After being stirred at r.t. for 1 h, the reaction mixture was evaporated under reduced pressure and crude **S3** was purified with preparative HPLC to afford **S3** (11.1 mg, 7.04 μmol as 2TFA salt, 7.0 %, 2 steps) and half crude **S4** was purified with preparative HPLC to afford **S4** (8.16 mg, 5.29 μmol as 2TFA salt, 8.1 %, 2 steps) as off-white solid after lyophilization.
S3: MALDI-TOFMS (CHCA) *m/z Calcd*: 1350.65 [M+H]+, Found: 1906.84; Retention time: 42.6 min.
S4: MALDI-TOFMS (CHCA) *m/z Calcd*: 1306.62 [M+H]+, Found: 1350.53; Retention time: 41.8 min.

To stirred solution of **S3** (3.0 mg, 1.90 μmol as 2TFA salt, 1.0 eq.) or **S4** (3.0 mg, 1.94 μmol as 2TFA salt, 1.0 eq.), mBAHA-COOH[1] (1.09 mg, 1.90 μmol, 1.0 eq.; 1.11 mg, 1.94 μmol, 1.0 eq), PyAOP (3.96 mg, 7.60 μmol, 4.0 eq.; 4.05 mg, 7.76 μmol, 4.0 eq.) in 94.8 or 97.0 μL DMF, DIEA (1.94 µL, 11.4 μmol, 6.0 eq.; 1.97 μL, 11.6 μmol, 6.0 eq.) was added. After being stirred at r.t. for 1 h, the reaction mixture was purified with preparative HPLC to afford **1** (PIP-AEA-BAHA, 1.01 mg, 0.526 μmol as TFA salt, 27.7%) and **3** (PIP-Gly-BAHA, 1.04 mg, 0.514 μmol as TFA salt, 26.5%) as white solid after lyophilization. Obtained PIP-Linker-BAHA was detected by MALDI-TOFMS.
PIP-AEA-BAHA (**1**): MALDI-TOFMS (CHCA) *m/z Calcd*: 1906.94 [M+H]+, Found: 1906.84; Retention time: 44.7 min.
PIP-Gly-BAHA (**3**): MALDI-TOFMS (CHCA) *m/z Calcd*: 1862.91 [M+H]+, Found: 1862.97; Retention time: 44.2 min.

Methods for biochemical experiments

PCR amplification and mutagenesis

PCR amplification was conducted either by Phusion High-Fidelity DNA Polymerase (Thermo Fisher) or KOD One PCR Master Mix (Toyobo), following the manufacturer's protocol. The sequence of PCR primers is summarized in Table S1. After purification by PCR Clean-Up Mini Kit (Favorgen) following the protocol provided by the manufacturer, a purified DNA fragment was used for further processes.

The N5_FWD.HindIII/PflMI and N5_REV.XhoI/BstXI primers were used with the pUC-SI-12-601-WT plasmid[2] as a template to amplify the N5 601 sequence, incorporating HindIII/PflMI and XhoI/BstXI restriction sites. The amplified N5 601 sequence was subsequently inserted into the pcDNA5/TO vector. The resulting plasmid, pSAK880, was then used as the template for further mutagenesis.

Mutagenesis PCR was performed using the PrimeSTAR Mutagenesis Basal Kit (Takara) according to the manufacturer's protocol. After transformation into DH5α cells, plasmid purification was carried out using the QIAprep Spin Miniprep Kit (QIAGEN) following the manufacturer's instructions. The purified plasmid was then used for subsequent processes.

The PIP-binding site sequence ATTACT in pSAK880 was mutagenized to AGCCGT using the primers N5_PIP-binding site mut_1_FWD and N5_PIP-binding site mut_1_REV. The resulting plasmid, pSAK881, was used as a template for additional mutagenesis.
The PIP-binding site sequence TATATACAT in pSAK881 was further mutagenized to GCTGCACAG using the primers N5_PIP-binding site mut_2_FWD and N5_PIP-binding site mut_2_REV. The resulting plasmid, pSAK882, served as the template for further mutagenesis and PCR amplification.

To construct DNA containing the PIP-binding site sequence AGTAAT/ATTACT for the N1 nucleosome, PCR amplification of **DNA1** was performed using pSAK882 as the template and N1_Ac(1)_FWD.PflMI and N1_Ac(1)_REV.BstXI as the primers. Following purification, **DNA1** was excised from the plasmid using PflMI and BstXI digestion.

DNA1:
5'-
ATGGCCGGATCCCCATCAGCAGTAATGGGCCGAGGCCGCTCAATTGGTCGTAGACA
GCTCTAGCACCGCTTAAACGCACGTACGCGCTGTCCCCCGCGTCCTAACCGCCAAG
GGGAGCCGTCCCTAGTCTCCAGGCACGTGTCAGCCATTACTGGCGATTTCCATTGCC
GCCACGATT
* PIP-binding site

Two plug-and-play sites (CTCGTG) for dU-39 or dU-49 insertion were added to the pSAK882 plasmid using the primers N5_Plug-and-play site dU-39 mut_FWD and N5_Plug-and-play site dU-39 mut_REV, or N5_Plug-and-play site dU-49 mut_FWD and N5_Plug-and-play site dU-49 mut_REV, respectively. The resulting plasmids, pSAK883 (dU-39) and pSAK885 (dU-49), were used as templates for further mutagenesis and PCR amplification.

To construct DNA containing the plug-and-play sites (CTCGTG) for dU-39 for the N1 nucleosome and the PIP-binding site sequence AGTAAT/ATTACT for the N1 nucleosome, PCR amplification of **DNA2** was carried out using the pSAK883 plasmid as the template and N1_dU(1)Ac(1)_dU-39_FWD.PflMI and N1_Ac(1)_REV.BstXI as the primers. Following purification, **DNA2** was excised from the plasmid using PflMI and BstXI digestion.

DNA2:
5’-
ATGGCCGGATCCCCATCAGCAGTAATGGGCCGAGGCTCGTGCCTTGG***T***CCTCGTGA
GCTCTAGCACCGCTTAAACGCACGTACGCGCTGTCCCCCGCGTCCTAACCGCCAAG
GGGAGCCGTCCCTAGTCTCCAGGCACGTGTCAGCCATTACTGGCGATTTCCATTGCC
GCCACGATT
* PIP-binding site, Plug-and-play site (Nb.BssSI), ***dU-39***

To construct DNA containing the plug-and-play sites (CTCGTG) for dU-49 for the N1 nucleosome and the PIP-binding site sequence AGTAAT/ATTACT for the N1 nucleosome, PCR amplification of **DNA3** was performed using the pSAK885 plasmid as the template and N1_dU(1)Ac(1)_dU-49_FWD.PflMI and N1_Ac(1)_REV.BstXI as the primers. Following purification, **DNA3** was excised from the plasmid using PflMI and BstXI digestion.

DNA3:
5’-
ATGGCCGGATCCCCATCAGCAGTAATGGGCCTCGTGC***C***CTCAATTGCTCGTGGACA
GCTCTAGCACCGCTTAAACGCACGTACGCGCTGTCCCCCGCGTCCTAACCGCCAAG
GGGAGCCGTCCCTAGTCTCCAGGCACGTGTCAGCCATTACTGGCGATTTCCATTGCC
GCCACGATT
* PIP-binding site, Plug-and-play site (Nb.BssSI), ***dU-49***

PCR amplification of DNA for the N2 nucleosome was carried out using the pSAK882 plasmid as the template and N2_FWD.BstXI and N2_REV.DraIII as the primers. Following purification, **DNA4** was excised from the plasmid using BstXI and DraIII digestion.

DNA4:
5’-
TTGGCGGATCCCCTGGAGAATCCCGGTGCCGAGGCCGCTCAATTGGTCGTAGACAG
CTCTAGCACCGCTTAAACGCACGTACGCGCTGTCCCCCGCGTCCTAACCGCCAAGG
GGAGCCGTCCCTAGTCTCCAGGCACGTGTCAGAGCTGCACAGCCTGTTCCATTGCC
GCACTAC

PCR amplification of DNA for the N3 nucleosome was performed using pSAK882 plasmid as template and N3_FWD.DraIII and N3_REV.BstEII as the primers. After purification, **DNA5** was digested from plasmid using DraIII and BstEII.

DNA5:
5’-

GTGATCCGGATCCCCTGGAGAATCCCGGTGCCGAGGCCGCTCAATTGGTCGTAGAC
AGCTCTAGCACCGCTTAAACGCACGTACGCGCTGTCCCCCGCGTCCTAACCGCCAA
GGGGAGCCGTCCCTAGTCTCCAGGCACGTGTCAGAGCTGCACAGCCTGTTCCATTG
CCGG

PCR amplification of DNA containing the plug-and-play sites (CTCGTG) for dU-39 for the N3 nucleosome was performed using pSAK883 plasmid as template and N3_FWD.DraIII and N3_REV.BstEII as the primers. Following purification, **DNA6** was excised from the plasmid using DraIII and BstEII digestion.

DNA6:
5’-
GTGATCCGGATCCCCTGGAGAATCCCGGTGCCGAGGCTCGTGCCTTGG***T***CCTCGTG
AGCTCTAGCACCGCTTAAACGCACGTACGCGCTGTCCCCCGCGTCCTAACCGCCAA
GGGGAGCCGTCCCTAGTCTCCAGGCACGTGTCAGAGCTGCACAGCCTGTTCCATTG
CCGG
* Plug-and-play site (Nb.BssSI), ***dU-39***

PCR amplification of DNA for the N4 nucleosome was carried out using the pSAK882 plasmid as the template and N4_FWD.BstEII and N4_REV.AvaI as the primers. Following purification, **DNA7** was excised from the plasmid using BstEII and AvaI digestion.

DNA7:
5’-
GTAACCGCATCCGGATCCCCTGGAGAATCCCGGTGCCGAGGCCGCTCAATTGGTCG
TAGACAGCTCTAGCACCGCTTAAACGCACGTACGCGCTGTCCCCCGCGTCCTAACC
GCCAAGGGGAGCCGTCCCTAGTCTCCAGGCACGTGTCAGAGCTGCACAGCCTGTTC
CATTGCCGC

DNA2, **DNA4**, **DNA5**, and **DNA7** were ligated together using T4 DNA ligase (Wako Nippon Gene) to obtain the **DNA for the tetra-nucleosome dU(1)Ac(1) dU-39**. This ligated product was then inserted into the pUC19 vector to generate the pSAK884 plasmid.

DNA for the tetra-nucleosome dU(1)Ac(1) dU-39:
5’-
ATGGCCGGATCCCCATCAGCAGTAATGGGCCGAGGCTCGTGCCTTGG***T***CCTCGTGA
GCTCTAGCACCGCTTAAACGCACGTACGCGCTGTCCCCCGCGTCCTAACCGCCAAG
GGGAGCCGTCCCTAGTCTCCAGGCACGTGTCAGCCATTACTGGCGATTTCCATTGCC
GCCACGATTTTGGCGGATCCCCTGGAGAATCCCGGTGCCGAGGCCGCTCAATTGGT
CGTAGACAGCTCTAGCACCGCTTAAACGCACGTACGCGCTGTCCCCCGCGTCCTAA
CCGCCAAGGGGAGCCGTCCCTAGTCTCCAGGCACGTGTCAGAGCTGCACAGCCTGT
TCCATTGCCGCACTACGTGATCCGGATCCCCTGGAGAATCCCGGTGCCGAGGCCGC
TCAATTGGTCGTAGACAGCTCTAGCACCGCTTAAACGCACGTACGCGCTGTCCCCC
GCGTCCTAACCGCCAAGGGGAGCCGTCCCTAGTCTCCAGGCACGTGTCAGAGCTGC
ACAGCCTGTTCCATTGCCGGGTAACCGCATCCGGATCCCCTGGAGAATCCCGGTGC
CGAGGCCGCTCAATTGGTCGTAGACAGCTCTAGCACCGCTTAAACGCACGTACGCG

CTGTCCCCCGCGTCCTAACCGCCAAGGGGAGCCGTCCCTAGTCTCCAGGCACGTGT
CAGAGCTGCACAGCCTGTTCCATTGCCGC
* PIP-binding site, Plug-and-play site (Nb.BssSI), ***dU-39***

DNA3, **DNA4**, **DNA5**, and **DNA7** were ligated together using T4 DNA ligase (Wako Nippon Gene) to obtain the **DNA for the tetra-nucleosome dU(1)Ac(1) dU-49**. This ligated product was then inserted into the pUC19 vector to generate the pSAK886 plasmid.

DNA for the tetra-nucleosome dU(1)Ac(1) dU-49:
5’-
ATGGCCGGATCCCCATCAGCAGTAATGGGCCTCGTGC***C***CTCAATTGCTCGTGGACA
GCTCTAGCACCGCTTAAACGCACGTACGCGCTGTCCCCCGCGTCCTAACCGCCAAG
GGGAGCCGTCCCTAGTCTCCAGGCACGTGTCAGCCATTACTGGCGATTTCCATTGCC
GCCACGATTTTGGCGGATCCCCTGGAGAATCCCGGTGCCGAGGCCGCTCAATTGGT
CGTAGACAGCTCTAGCACCGCTTAAACGCACGTACGCGCTGTCCCCCGCGTCCTAA
CCGCCAAGGGGAGCCGTCCCTAGTCTCCAGGCACGTGTCAGAGCTGCACAGCCTGT
TCCATTGCCGCACTACGTGATCCGGATCCCCTGGAGAATCCCGGTGCCGAGGCCGC
TCAATTGGTCGTAGACAGCTCTAGCACCGCTTAAACGCACGTACGCGCTGTCCCCC
GCGTCCTAACCGCCAAGGGGAGCCGTCCCTAGTCTCCAGGCACGTGTCAGAGCTGC
ACAGCCTGTTCCATTGCCGGGTAACCGCATCCGGATCCCCTGGAGAATCCCGGTGC
CGAGGCCGCTCAATTGGTCGTAGACAGCTCTAGCACCGCTTAAACGCACGTACGCG
CTGTCCCCCGCGTCCTAACCGCCAAGGGGAGCCGTCCCTAGTCTCCAGGCACGTGT
CAGAGCTGCACAGCCTGTTCCATTGCCGC
* PIP-binding site, Plug-and-play site (Nb.BssSI), ***dU-49***

DNA1, **DNA4**, **DNA6**, and **DNA7** were ligated together using T4 DNA ligase (Wako Nippon Gene) to obtain the **DNA for the tetra-nucleosome dU(3)Ac(1) dU-39**. This ligated product was then inserted into the pUC19 vector to generate the pSAK887 plasmid.

DNA for the tetra-nucleosome dU(3)Ac(1) dU-39:
5’-
ATGGCCGGATCCCCATCAGCAGTAATGGGCCGAGGCCGCTCAATTGGTCGTAGACA
GCTCTAGCACCGCTTAAACGCACGTACGCGCTGTCCCCCGCGTCCTAACCGCCAAG
GGGAGCCGTCCCTAGTCTCCAGGCACGTGTCAGCCATTACTGGCGATTTCCATTGCC
GCCACGATTTTGGCGGATCCCCTGGAGAATCCCGGTGCCGAGGCCGCTCAATTGGT
CGTAGACAGCTCTAGCACCGCTTAAACGCACGTACGCGCTGTCCCCCGCGTCCTAA
CCGCCAAGGGGAGCCGTCCCTAGTCTCCAGGCACGTGTCAGAGCTGCACAGCCTGT
TCCATTGCCGCACTACGTGATCCGGATCCCCTGGAGAATCCCGGTGCCGAGGCTCG
TGCCTTGG***T***CCTCGTGAGCTCTAGCACCGCTTAAACGCACGTACGCGCTGTCCCCCG
CGTCCTAACCGCCAAGGGGAGCCGTCCCTAGTCTCCAGGCACGTGTCAGAGCTGCA
CAGCCTGTTCCATTGCCGGGTAACCGCATCCGGATCCCCTGGAGAATCCCGGTGCC
GAGGCCGCTCAATTGGTCGTAGACAGCTCTAGCACCGCTTAAACGCACGTACGCGC
TGTCCCCCGCGTCCTAACCGCCAAGGGGAGCCGTCCCTAGTCTCCAGGCACGTGTC
AGAGCTGCACAGCCTGTTCCATTGCCGC
* PIP-binding site, Plug-and-play site (Nb.BssSI), ***dU-39***

To construct DNA containing the plug-and-play sites (CTCGTG) for dU-39 and PIP-binding sites for the N3 nucleosome, PCR amplification of **DNA8** was performed using **DNA2** as template and N3_site change_FWD.DraIII and N3_site change_REV.BstEII as primers.

DNA8:
5’-
GTGATCCGGATCCCCATCAGCAGTAATGGGCCGAGGCTCGTGCCTTGG***T***CCTCGTG
AGCTCTAGCACCGCTTAAACGCACGTACGCGCTGTCCCCCGCGTCCTAACCGCCAA
GGGGAGCCGTCCCTAGTCTCCAGGCACGTGTCAGCCATTACTGGCGATTTCCATTG
CCGG
* PIP-binding site, Plug-and-play site (Nb.BssSI), ***dU-39***

To construct DNA for the N1 nucleosome, PCR amplification of **DNA9** was performed using **DNA5** as template and N1_site change_FWD.PflMI and N1_site change_REV.BstXI as primers.

DNA9:
5’-
ATGGCCGGATCCCCTGGAGAATCCCGGTGCCGAGGCCGCTCAATTGGTCGTAGACA
GCTCTAGCACCGCTTAAACGCACGTACGCGCTGTCCCCCGCGTCCTAACCGCCAAG
GGGAGCCGTCCCTAGTCTCCAGGCACGTGTCAGAGCTGCACAGCCTGTTCCATTGC
CGCCACGATT

To construct DNA containing PIP-binding sites for the N3 nucleosome, PCR amplification of **DNA10** was performed using **DNA1** as template and N3_site change_FWD.DraIII and N3_site change_REV.BstEII as primers.

DNA10:
5’-
GTGATCCGGATCCCCATCAGCAGTAATGGGCCGAGGCCGCTCAATTGGTCGTAGAC
AGCTCTAGCACCGCTTAAACGCACGTACGCGCTGTCCCCCGCGTCCTAACCGCCAA
GGGGAGCCGTCCCTAGTCTCCAGGCACGTGTCAGCCATTACTGGCGATTTCCATTG
CCGG
* PIP-binding site

To construct DNA containing the plug-and-play sites (CTCGTG) for dU-39 for the N1 nucleosome, PCR amplification of **DNA11** was performed using **DNA6** as template and N1_site change_FWD.PflMI and N1_site change_REV.BstXI as the primers.

DNA11:
5’-
ATGGCCGGATCCCCTGGAGAATCCCGGTGCCGAGGCTCGTGCCTTGG***T***CCTCGTGA
GCTCTAGCACCGCTTAAACGCACGTACGCGCTGTCCCCCGCGTCCTAACCGCCAAG
GGGAGCCGTCCCTAGTCTCCAGGCACGTGTCAGAGCTGCACAGCCTGTTCCATTGC
CGCCACGATT
* Plug-and-play site (Nb.BssSI), ***dU-39***

To construct the **DNA for the tetra-nucleosome dU(3)Ac(3) dU-39**, **DNA9** was inserted into the pSAK887 plasmid to obtain the pSAK888 plasmid. Subsequently, **DNA8** was inserted into the pSAK888 plasmid to generate the pSAK889 plasmid.

DNA for the tetra-nucleosome dU(3)Ac(3) dU-39:

5’-
ATGGCCGGATCCCCTGGAGAATCCCGGTGCCGAGGCCGCTCAATTGGTCGTAGACA
GCTCTAGCACCGCTTAAACGCACGTACGCGCTGTCCCCCGCGTCCTAACCGCCAAG
GGGAGCCGTCCCTAGTCTCCAGGCACGTGTCAGAGCTGCACAGCCTGTTCCATTGC
CGCCACGATTTTGGCGGATCCCCTGGAGAATCCCGGTGCCGAGGCCGCTCAATTGG
TCGTAGACAGCTCTAGCACCGCTTAAACGCACGTACGCGCTGTCCCCCGCGTCCTA
ACCGCCAAGGGGAGCCGTCCCTAGTCTCCAGGCACGTGTCAGAGCTGCACAGCCTG
TTCCATTGCCGCACTACGTGATCCGGATCCCCATCAGCAGTAATGGGCCGAGGCTC
GTGCCTTGG***T***CCTCGTGAGCTCTAGCACCGCTTAAACGCACGTACGCGCTGTCCCCC
GCGTCCTAACCGCCAAGGGGAGCCGTCCCTAGTCTCCAGGCACGTGTCAGCCATTA
CTGGCGATTTCCATTGCCGGGTAACCGCATCCGGATCCCCTGGAGAATCCCGGTGC
CGAGGCCGCTCAATTGGTCGTAGACAGCTCTAGCACCGCTTAAACGCACGTACGCG
CTGTCCCCCGCGTCCTAACCGCCAAGGGGAGCCGTCCCTAGTCTCCAGGCACGTGT
CAGAGCTGCACAGCCTGTTCCATTGCCGC

* PIP-binding site, Plug-and-play site (Nb.BssSI), ***dU-39***

To construct the **DNA for the tetra-nucleosome dU(1)Ac(3) dU-39**, **DNA11** was inserted into pSAK887 plasmid to obtain pSAK890 plasmid, then **DNA10** was inserted into pSAK890 plasmid to obtain pSAK891 plasmid.

DNA for the tetra-nucleosome dU(1)Ac(3) dU-39:

5’-
ATGGCCGGATCCCCTGGAGAATCCCGGTGCCGAGGCTCGTGCCTTGG***T***CCTCGTGA
GCTCTAGCACCGCTTAAACGCACGTACGCGCTGTCCCCCGCGTCCTAACCGCCAAG
GGGAGCCGTCCCTAGTCTCCAGGCACGTGTCAGAGCTGCACAGCCTGTTCCATTGC
CGCCACGATTTTGGCGGATCCCCTGGAGAATCCCGGTGCCGAGGCCGCTCAATTGG
TCGTAGACAGCTCTAGCACCGCTTAAACGCACGTACGCGCTGTCCCCCGCGTCCTA
ACCGCCAAGGGGAGCCGTCCCTAGTCTCCAGGCACGTGTCAGAGCTGCACAGCCTG
TTCCATTGCCGCACTACGTGATCCGGATCCCCATCAGCAGTAATGGGCCGAGGCCG
CTCAATTGGTCGTAGACAGCTCTAGCACCGCTTAAACGCACGTACGCGCTGTCCCC
CGCGTCCTAACCGCCAAGGGGAGCCGTCCCTAGTCTCCAGGCACGTGTCAGCCATT
ACTGGCGATTTCCATTGCCGGGTAACCGCATCCGGATCCCCTGGAGAATCCCGGTG
CCGAGGCCGCTCAATTGGTCGTAGACAGCTCTAGCACCGCTTAAACGCACGTACGC
GCTGTCCCCCGCGTCCTAACCGCCAAGGGGAGCCGTCCCTAGTCTCCAGGCACGTG
TCAGAGCTGCACAGCCTGTTCCATTGCCGC

* PIP-binding site, Plug-and-play site (Nb.BssSI), ***dU-39***

Preparation of DNA for tetra-nucleosome reconstitution

Plasmids containing DNA for tetra-nucleosome (pSAK884, 886, 887, 889, 891) were digested using PflMI (NEB) and AvaI (NEB), and DNA for tetra-nucleosome was purified using gel purification kit (FAVORGEN, FavorPrep GEL Purification Mini Kit) and stored at -30 °C.

Preparation of DNA for mono-nucleosome reconstitution

DNA for the mono-nucleosome was prepared by PCR amplification using the pSAK884 or pSAK886 plasmids as templates and N1_Mono-DNA_FWD and N1_Mono-DNA_REV as the primers. The PCR products were purified by phenol-chloroform extraction and ethanol precipitation. The purified DNA was redissolved in TE buffer (10 mM Tris-HCl, 1 mM EDTA, pH 8.0) and further purified using a Model 491 Prep Cell (BIO-RAD) with a 7 cm, 6% native PAGE (29:1 acrylamide:bisacrylamide, 0.2×TBE buffer) column and an AKTA system (GE Healthcare). DNA in the collected fractions were assessed by 6% native PAGE electrophoresis (29:1 acrylamide:bisacrylamide, 0.2×TBE buffer, 200 V, 20 min). Fractions containing pure DNA for mono-nucleosome were collected, concentrated by ultrafiltration (Millipore, Amicon® Ultra-15 mL, Centrifugal Filters Ultracel®-3K), and stored at -30 °C.

DNA for the mono-nucleosome dU(1)Ac(1) dU-39:

5'-
ATGGCCGGATCCCCATCAGCAGTAATGGGCCGAGGCTCGTGCCTTGG***T***CCTCGTGA
GCTCTAGCACCGCTTAAACGCACGTACGCGCTGTCCCCCGCGTCCTAACCGCCAAG
GGGAGCCGTCCCTAGTCTCCAGGCACGTGTCAGCCATTACTGGCGATTTCCATTGCC
GCCACGATT

* Primer N1_Mono-DNA_FWD/REV, PIP-binding site, Plug-and-play site (Nb.BssSI), ***dU-39***

DNA for the mono-nucleosome dU(1)Ac(1) dU-49:

5'-
ATGGCCGGATCCCCATCAGCAGTAATGGGCCTCGTGC***C***CTCAATTGCTCGTGGACA
GCTCTAGCACCGCTTAAACGCACGTACGCGCTGTCCCCCGCGTCCTAACCGCCAAG
GGGAGCCGTCCCTAGTCTCCAGGCACGTGTCAGCCATTACTGGCGATTTCCATTGCC
GCCACGATT

* Primer N1_Mono-DNA_FWD/REV, PIP-binding site, Plug-and-play site (Nb.BssSI), ***dU-49***

Histone preparation and octamer refolding

Human histones H2A, H2B, H3.1, and H4 were expressed and purified according to established protocols.[3,4] Histone octamers were refolded using standard protocol,[5] and purified by size exclusion chromatography using a SuperDex 200 10/300 GL column (GE Lifesciences, Boston, MA). The purified histone octamer was stored in octamer buffer (2 M NaCl, 5 mM BME, 0.2 mM PMSF, 1 mM EDTA, 10 mM HEPES, pH 7.8).

Tetra-nucleosome reconstitution

The reconstitution of nucleosome arrays generally followed the protocol described previously,[5] with some modifications. For small-scale reconstitution, DNA for tetra-nucleosome was mixed with varying molar ratios of refolded histone octamers in a 15 μL reaction mixture containing 2 M NaCl at 4 °C, with the final DNA concentration in each sample set to 0.1 μg/μL. The reaction

mixture was then transferred to a 3.5k MWCO Slide-A- Lyzer MINI dialysis unit (Thermo Fisher Scientific, Waltham, MA) and floated over 500 mL of pre-cooled nucleosome high salt (HS) buffer (2 M NaCl, 10 mM HEPES, pH 7.8). Over a period of approximately 23 h at 4 °C, the HS buffer was exchanged with 2.0 L low salt (LS) buffer (25 mM NaCl, 10 mM HEPES, pH 7.8) using a peristaltic pump (Atto, WSP-3300 PeristaQuantumPump) set to 1.20 mL/min. Once the buffer exchange was completed, the reconstitution mixtures were spun at 13,000 rpm for 20 min to remove any aggregates and the supernatant was collected and stored at 4 °C. For large-scale reconstitution, the volume for each dialysis unit was limited to no more than 50 μL. The reconstituted arrays were analyzed by 6% native PAGE (59:1 acrylamide:bisacrylamide, 0.2×TBE buffer) and stained with ethidium bromide. The concentration was determined by measuring the absorbance at 260 nm in 0.1 M NaOH, as previously reported.[5]

Mono-nucleosome reconstitution

Mono-nucleosomes were reconstituted using the same salt dialysis method described above. The reconstituted mono-nucleosomes were analyzed by 6% native PAGE (59:1 acrylamide:bisacrylamide, 0.2×TBE buffer) and stained with ethidium bromide. The concentrations were determined by measuring the absorbance at 260 nm in 0.1 M NaOH as previously reported.[5]

Nucleosome acetylation

Tetra-nucleosome acetylation by PIP-AEA-BAHA (1), PIP-Gly-BAHA (3) or TMP-BAHA (4)

16 ng/μL tetra-nucleosome was incubated with 5 μM PIP-AEA-BAHA (**1**), PIP-Gly-BAHA (**3**) or TMP-BAHA (**4**)[1], 500 μM acetyl donor (**2**)[1] in 150 mM NaCl, 50 mM Tris-HCl buffer at 37 °C for 16 h. H3K56 acetylation was detected by western blot analysis as follows. Samples were prepared by adding sample buffer (5×SB (250 mM Tris-HCl pH 6.8, 30% glycerol v/v, 10% SDS w/v, 0.05% bromophenol blue w/v): 1 M DTT = 2 : 1) to a reaction mixture, followed by boiling at 95 °C for 5 minutes. Proteins were separated by 15% SDS-PAGE and transferred to Immobilon PSQ PVDF membranes (Millipore). For blocking, 5% skim milk (Nacalai) in TBS containing 0.1% Tween-20 (Santa Cruz) was used. The following antibodies were used: Primary antibodies: H3K56Ac (Active Motif, Cat# 39281, Lot# 14013003, 1:3000); Secondary antibodies: Anti-rabbit IgG, HRP-linked Antibody (Cell Signaling Technology, Cat# 7074S, Lot# 27, 1:5000). Chemiluminescence was generated using Luminata Forte HRP substrate (Millipore) and detected with the Amersham ImageQuant 800 (Cytiva). For protein staining, gels were stained with Oriole Fluorescent Gel Stain (BIO-RAD).

Tetra-nucleosome acetylation by PIP-AEA-BAHA (1) and sirtuin

16 ng/μL tetra-nucleosome was incubated with 5 μM PIP-AEA-BAHA (**1**), 500 μM acetyl donor (**2**) in 150 mM NaCl, 50 mM Tris-HCl buffer at 37 °C for 16 h. Then, Sirt3 (15 ng/μL in final concentration, ACTIVE MOTIF (102-399), Cat#31529) and/or Sirt7 (5.0 ng/μL in final concentration, purified as previously described[6]) was reacted in buffer containing 20 mM NAD^+, 137 mM NaCl, 2.7 mM KCl, 1 mM $MgCl_2$, and 25 μM $ZnSO_4$ at 30 °C for 3 h. Acetyl-lysines were detected by western blot analysis as follows. Samples were prepared as described above. Proteins were separated by 15% SDS-PAGE and transferred to Immobilon PSQ PVDF membranes (Millipore). For blocking, 5% skim milk (Nacalai) in TBS containing 0.1% Tween-

20 (Santa Cruz) was used. The following antibodies were used: Primary antibodies: Acetylated-Lysine Antibody (Cell Signaling Technology, Cat# 9441L, Lot# 16, 1:1000); Secondary antibodies: Anti-rabbit IgG, HRP-linked Antibody (Cell Signaling Technology, Cat# 7074S, Lot# 27, 1:5000). Chemiluminescence was generated using Luminata Forte HRP substrate (Millipore) and detected with the Amersham ImageQuant 800 (Cytiva). For protein staining, gels were stained with Oriole Fluorescent Gel Stain (BIO-RAD).

Sample preparation for LC–MS/MS analysis of tetra-nucleosome

Acetylated tetra-nucleosomes were purified and concentrated using ultrafiltration (Millipore, Amicon® Ultra-0.5 mL, Centrifugal Filters Ultracel®-30K) and separated by electrophoresis on a 6% native PAGE gel (59:1 acrylamide:bisacrylamide, 0.2×TBE buffer). In-gel digestion was then performed with slight modifications to previously reported methods.[7,8,9] Nucleosomes were visualized by ethidium bromide staining, and the corresponding gel bands were excised and incubated in 1% SDS at 4 °C for overnight. After washing with 100mM NH_4HCO_3 and acetonitrile, DNA was digested with DNase I for 30 minutes at 37 °C, followed by additional washes with 100 mM NH_4HCO_3 and acetonitrile. Samples were suspended in 180 μL of 100 mM aqueous ammonium bicarbonate, and 180 μL of propionic anhydride solution (methanol/propionic anhydride, 3:1 (v/v)) was added. The pH was adjusted to 8.0 by adding 162 μL of 28% aqueous ammonium hydroxide. The mixture was stirred at r.t. for 30 min, and the solvents were subsequently removed, followed by washing with 100 mM NH_4HCO_3 and acetonitrile. For DTT reduction, the gel was incubated with 10 mM DTT in 100 mM NH_4HCO_3 at 56 °C for 1 h. After solvent removal, thiol groups were protected by reacting with 55 mM iodoacetamide in 100 mM NH_4HCO_3 at r.t. for 45 min, followed by additional washes with NH_4HCO_3 and acetonitrile. The peptide digestion was carried out in gel using 10 ng/μL trypsin gold in 50 mM NH_4HCO_3 at 37 ^{o}C overnight. Peptides were extracted from the gel step-by-step using MilliQ water, 0.1% TFA in 60% CH_3CN, 0.1% TFA in 80% CH_3CN, and 0.1% TFA in 100% CH_3CN. The extracted peptides were collected in the final extraction solution, concentrated using a speed vacuum, and dissolved in 0.1% formic acid. After centrifugation at 15,000 rpm for 10 minutes, the supernatant was used for LC-MS/MS detection. For LC-MS/MS analysis of N1 or N2–N4 nucleosomes, acetylated tetra-nucleosomes were purified and concentrated using ultrafiltration, followed by digestion with BstXI. The resulting fragments were separated on a 6% native PAGE gel (59:1 acrylamide:bisacrylamide, 0.2×TBE buffer). In-gel digestion and peptide extraction were performed as described above.

LC–MS/MS analysis

LC–MS/MS analyses were conducted using AB Sciex Triple TOF 4600 equipped with eksigent ekspert microLC 200. LC separation was conducted on a 3C18–CL–120 column (0.5 mm I.D. × 100 mm) under a linear gradient of 2–35% acetonitrile with 0.1% formic acid (v/v) against water with 0.1% formic acid (v/v) over 8 min at 40 ^{o}C, with a flow rate of 20 μL min^{-1}. A sample volume of 5 μL was injected for each run. The eluate was monitored by an on-line quadrupole time-of-flight mass spectrometer (ESI-Q-TOF MS) operated in positive ion mode. Each measurement was performed in duplicate. Data analysis was conducted using PeakView software (AB Sciex, version 1.2.0.3). Selected intense and/or characteristic MS/MS fragment ions from each precursor ion were used to extract chromatograms, based on both precursor and fragment ions, with a mass tolerance of 0.2 Da. The stoichiometry of lysine acetylation was calculated as the percentage of

the total peak area of the extracted ion chromatogram for acetylated peptides relative to the sum of peak areas for acetylated and propionylated peptides. Details of digestion enzymes, precursor ions, and MS/MS fragments used in LC–MS/MS analysis to determine lysine acetylation yields are provided below.

H3 GSHMARTKQTARKSTGGKAPRKQLATKAARKSAPATGGVKKPHRYRPGTVALREI RRYQ***K(56)***STELLIRKLPFQRLVREIAQDFKTDLRFQSSAVMALQEACEAYLVGLFEDT N LCAIHAKRVTIMPKDIQLARRIRGERA

Fragment	Sequence	Enzymes	Precursor ions (Pr / Ac)	MS/MS fragment ions
3–8	TKQTAR	Trypsin & GluC	380.72 / 373.71	*y*2, *y*3, *y*4, *y*5
9–17	KSTGGKAPR	Trypsin	507.29 / 500.28	*y*6, *y*7, *y*8, *y*9
18–26	KQLATKAAR	Trypsin & GluC	549.84 / 542.83	*y*5, *y*6, *y*7, *y*8
27–40	KSAPATGGVKKPHR	Trypsin & GluC	534.64 / 529.97	*y*5, *y*6, *y*7, *y*8
				*y*4
54–63	YQKSTELLIR	Trypsin	653.87 / 646.86	*b*3, *y*6, *y*7, *y*8
64–69	KLPFQR	Trypsin & GluC	422.76 / 415.75	*y*2, *y*3, *y*4, *y*5
73–83	EIAQDFKTDLR	Trypsin	696.36 / 689.35	*y*5, *y*6, *y*7, *y*8
106–116	DTNLCAIHAKR	Trypsin & GluC	649.34 / 642.33	*y*3, *y*4, *y*5, *y*6
117–128	VTIMPKDIQLAR	Trypsin	720.92 / 713.91	*b*3, *y*8, *y*9, *y*10

Mg^{2+}-promoted nucleosome sedimentation assay

The concentration of nucleosomes was adjusted to approximately 40 ng/μL dsDNA in LS buffer. A 9 μL aliquot of the acetylated nucleosomes in LS buffer was mixed with 1 μL of 40 mM $MgCl_2$ in LS buffer or LS buffer, and the mixture was incubated at 25 °C for 30 min. Following incubation, the samples were centrifuged at 15,000 rpm for 10 min at 25 °C. The A_{260} of the supernatant was measured using a NanoDrop Lite spectrophotometer (Thermo Fisher Scientific).

Plug-and-play for tetra-DNA

Insertion of dU into DNA for tetra-nucleosome followed the previously described protocol[2] with some modifications.

<u>Nicking</u>: DNA for tetra-nucleosome (20 μg, 43 pmol) was nicked using the nicking enzyme Nb.BssSI (130 units) in r3.1 buffer (NEB) at 37 °C for 2 h. A 2.5 μL aliquot was stored as a nicking sample for gel analysis.

<u>5' Phosphorylation of insert DNA with dU for ligation</u>: The single-stranded insert DNA (IDT) was phosphorylated by incubating with T4 polynucleotide kinase (5 units/100 pmol ssDNA, NEB) and 1 mM ATP (NEB) in T4 polynucleotide kinase buffer (NEB) at 37 °C for 30 min. The reaction products were purified by ethanol precipitation, and the 5' phosphorylated insert ssDNA with dU was redissolved in MilliQ water to a concentration of 100 pmol/μL and stored at -30 °C.

Capture and insertion: The nicking mixture was incubated with capture DNA (LNA, 252 pmol, 6.0 equivalents) and 5' phosphorylated insert DNA with dU (629 pmol, 15 equivalents) at 80 °C for 20 min, followed by cooling to 12 °C at a rate of –1 °C/min.
Ligation: Ligation was performed by incubating the mixture with T4 DNA ligase (1500 units) and ATP (Takara, final concentration of 5 mM) at 16 °C overnight. The ligation products were purified using a PCR purification kit (FAVORGEN®, FavorPrep™ PCR Clean-Up Mini Kit).

Plug-and-play for tetra-DNA with ^{32}P-label

Insertion of dU and [γ-^{32}P]-ATP into DNA for tetra-nucleosome or mono-nucleosome followed the previously described protocol[2] with some modifications.
Insert DNA labeling: Single-stranded DNA (800 pmol) was labeled by incubating with T4 polynucleotide kinase (40 units, NEB) and [γ-^{32}P]-ATP (J-RAM, final concentration 1 mM) in T4 polynucleotide kinase buffer at 37 °C for 80 min. The reaction products were purified by ethanol precipitation, and the ^{32}P-labeled DNA with dU was redissolved in MilliQ to a final concentration of 10 pmol/μL.
Nicking step: The nicking procedure was performed similarly to the non-labeled DNA. The nicking mixture was incubated with LNA (252 pmol, 6.0 equivalents) and insert DNA with dU (629 pmol total, consisting of 200 pmol ^{32}P-labeled insert DNA and 429 pmol non-labeled insert DNA, 15 equivalents) at 80 °C for 20 min. The mixture was then cooled to 12 °C at a rate of –1 °C/min.
Ligation step: The ligation step was identical to the non-labeled DNA.
Preparation and validation of DNA for tetra-nucleosomes or mono-nucleosome was shown in Figure S5.

^{32}P-Labeled nucleosome reconstitution and acetylation

^{32}P-labeled DNAs were reconstituted into nucleosomes with histone octamers using the same method as for non-labeled DNAs. Acetylated tetra-nucleosomes by ABECHS were purified and concentrated using ultrafiltration (Millipore, Amicon® Ultra-0.5 mL, Centrifugal Filters Ultracel®-30K). The final volume of the nucleosome solution was adjusted to 70 μL with the LS buffer. The quality of the nucleosomes was checked by 6% native PAGE (59:1 acrylamide:bisacrylamide, 0.2× TBE buffer). The quality of the nucleosomes was checked by 6% native PAGE (59:1 acrylamide:bisacrylamide, 0.2× TBE buffer) in Figures S6 and S7.

Base Excision Repair (BER) Assay: dU Cleavage by UDG/APE1

Reaction mixtures were prepared containing 4 nM of mono- or tetra-nucleosome in LS buffer (25 mM NaCl, 10 mM HEPES, pH 7.8) with 0.2 mM $MgCl_2$ (final concentration). Reactions were initiated by adding 0.1 nM each of UDG and APE1 (premixed), followed by incubation at 30°C. At specific time points (0 or 10 min), 7.5 μL aliquots were removed, and the reactions were quenched by adding 20 μL of a quenching buffer containing 1% (v/v) SDS, 10 mM $NaBH_4$, and 12.5 μL proteinase K (Takara, cat# 9034). The samples were then incubated at 37°C for 1 h. DNA was purified using phenol-chloroform extraction followed by ethanol precipitation. For substrates with dU inserted into the N3 nucleosome, the DNA was digested with DraIII and BstEII (20 units each) to excise the dU-containing 5'-[γ-^{32}P]-labeled oligonucleotide, followed by an additional round of phenol-chloroform extraction and ethanol precipitation. For substrates with dU inserted into the N1 nucleosome, the DNA was used directly without further digestion. The dried DNA was resuspended in 5 μL of r3.1 buffer (NEB) and combined with 8 μL of formamide (FUJIFILM

Wako, cat# 064-00423). The mixture was incubated at 95°C for 30 min. Samples were then analyzed on a 10% denaturing PAGE gel (19:1 acrylamide:bisacrylamide, 1× TBE buffer). The gel was pre-run at 300 V for 1 h before sample loading, and electrophoresis was performed at 300 V for 50 min. The gel was visualized using an Amersham Typhoon scanner (Cytiva).

The percentage of DNA cleavage after 10 min was calculated using the following equation:

Cleavage percentage (10 min) = (b − a) / (B + (b − a))

Where:

- **B**: Band intensity of uncleaved tetra- or mono-nucleosomal DNA at 10 min.
- **b**: Band intensity of cleaved DNA at 10 min.
- **a**: Band intensity of cleaved DNA at 0 min.

Band intensities were quantified using ImageQuant TL software (IQTL version 10.1, Cytiva). All experiments were performed in triplicate or greater for each substrate.

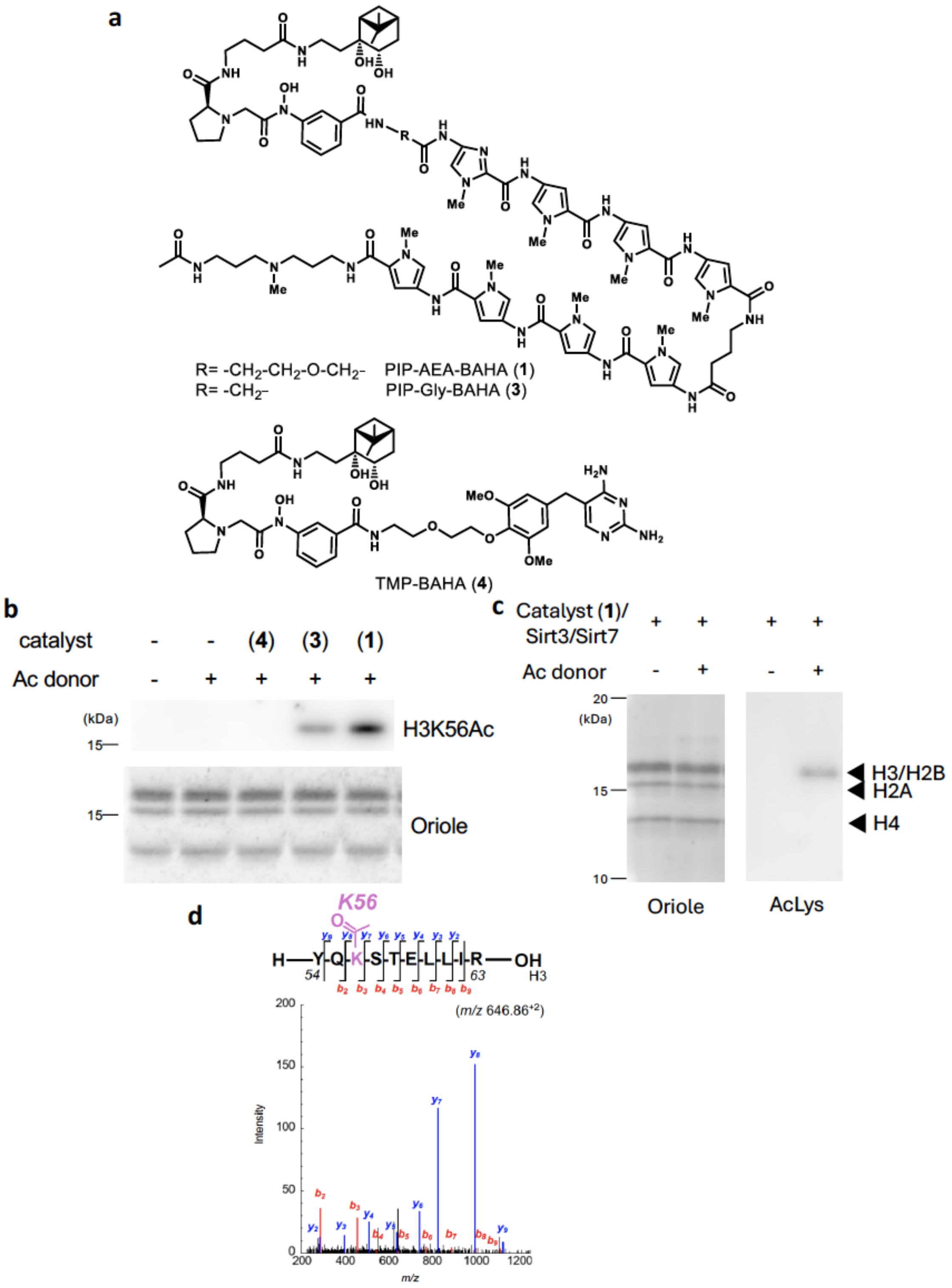

Figure S1. Abiotic catalyst optimization. (a) Structure of PIP-AEA-BAHA **1**, PIP-Gly-BAHA **3** and TMP-BAHA **4**. (b) Tetra-nucleosomes (16 ng/μL DNA) were treated with a catalyst **1**, **3**, or **4** (5 μM) and acetyl donor **2** (500 μM) in 150 mM NaCl, 50 mM Tris-HCl pH 7.5 buffer at 37 °C for 16 h, and acetylated lysines were detected by immunoblotting using H3K56Ac-specific antibodies. Histone proteins were visualized by oriole staining. Representative data from two

independent experiments are shown. (c) Tetra-nucleosomes (16 ng/μL DNA) were treated with PIP-AEA-BAHA **1** (5 μM) with/without acetyl donor **2** (500 μM) in 150 mM NaCl, 50 mM Tris-HCl pH 7.5 buffer at 37 °C for 16 h. Then, the acetylated nucleosome arrays were treated with Sirt3 (15 ng/μL) and/or Sirt7 (5 ng/μL) at 30 °C for 3 h. Acetylated lysines were detected by immunoblotting using AcLys antibodies. Histone proteins were visualized by oriole staining. Representative data from three independent experiments are shown. (d) Representative LC-MS/MS trace of the H3 Y54-R63 peptide (K56Ac) from the N1 sample in Figure 2e is shown

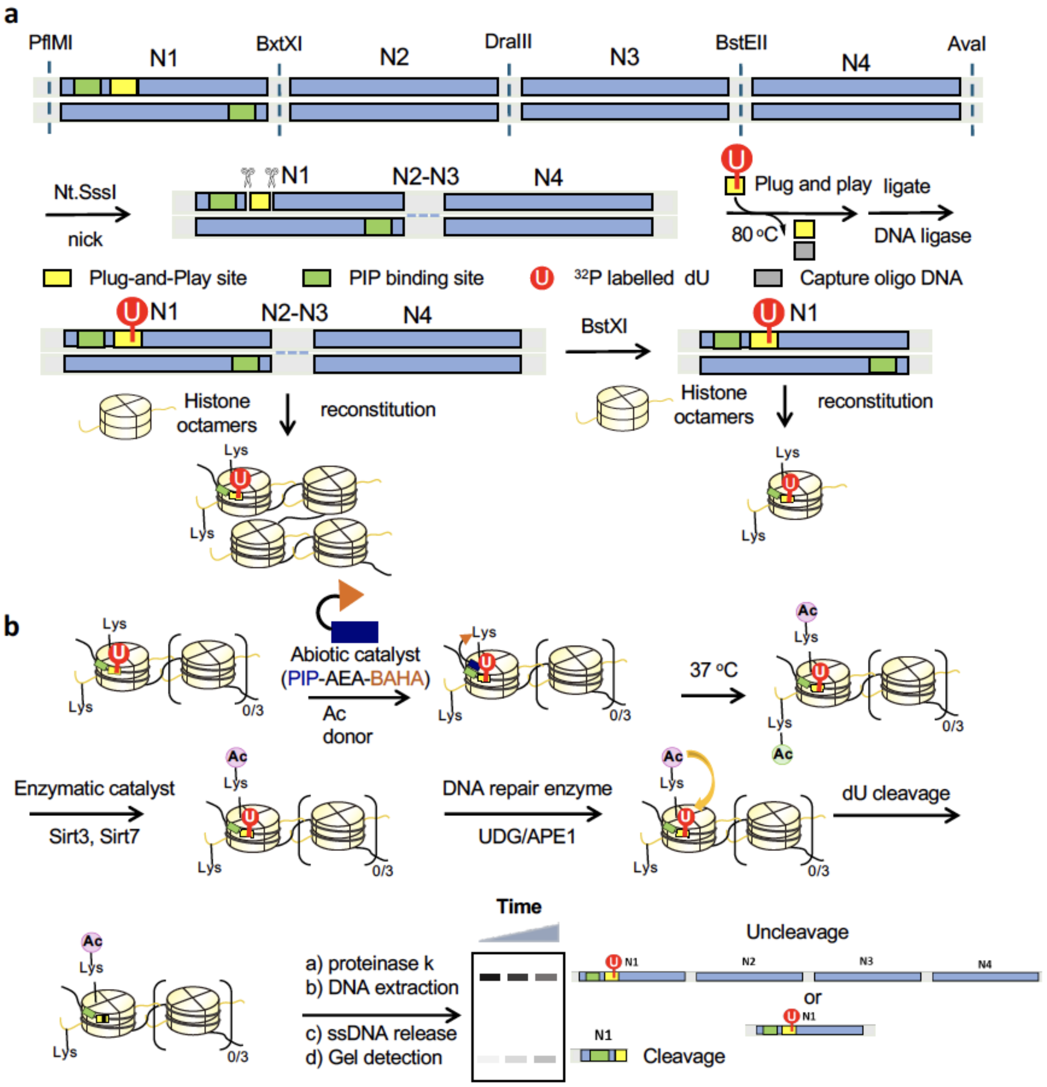

Figure S2. Scheme of reconstitution of tetra-nucleosome regioselectively containing a damage (dU) and PIP-binding site and DNA repair assay (a) The DNA sequence contained the plug-and-play site (yellow) and the PIP binding site (green). A ssDNA with 5'-^{32}P labeled uracil-DNA(dU) at position –39 or –49 was inserted into 601 DNA by the plug-and-play method. Briefly, both 3'- and 5'-ends of the plug-and-play site were nicked by Nb.BssSI. Then, the original ssDNA at the plug-and-play site was swapped by the ssDNA containing dU at 80 °C in the presence of capture oligo DNA (gray). After ligation by T4 DNA ligase, DNA with a four-repeat 601 sequence, regioselectively containing dU, was produced. After the linker DNA between N1 and N2 was digested by BstXI, DNA with a 601 sequence, regioselectively containing dU, was produced. The tetra-nucleosome or mono-nucleosome bearing dU was reconstituted by winding DNA around histone octamers. (b) The mono-nucleosome or tetra-nucleosome was treated with PIP-AEA-BAHA **1** catalyst and Ac-donor **2** at 37 °C for 16 h, followed by Sirt 3 and Sirt 7 at 30 °C for 3 h, to afford mono-nucleosome or tetra-nucleosomes containing dU at position –39 or –49 of N1 and H3K56Ac on N1. After Amicon wash, mono-nucleosome or tetra-nucleosomes were treated with DNA repair enzymes UDG and APE1 and the reaction was quenched at 0 or 10 min. The DNA repair efficacy was analyzed by 7 M urea denatured PAGE.

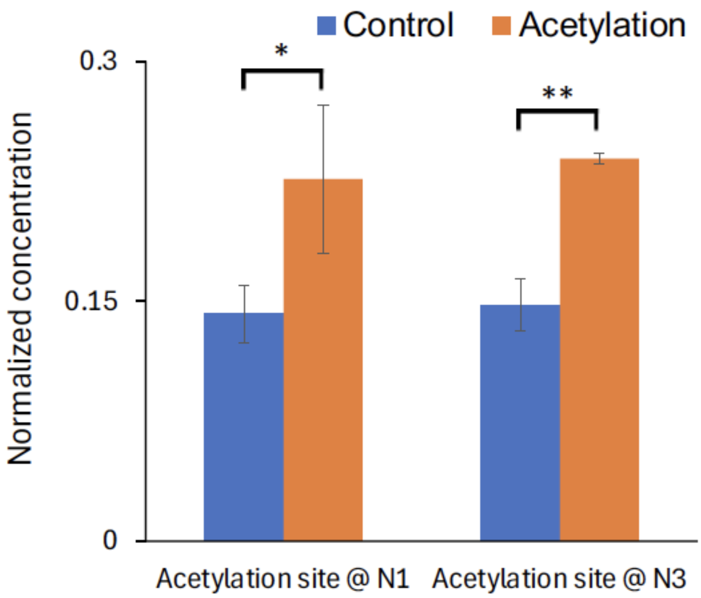

Figure S3. Sedimentation assay of tetra-nucleosome arrays. In this assay, tetra-nucleosomes with or without H3K56 acetylation at N1 or N3 under 0 mM or 4 mM $MgCl_2$ were incubated at room temperature for 30 min. The samples were then spun at 15000 rpm for 10 min at room temperature. The concentration of the supernatant was detected by nanodrop. The y-axis indicates the normalized concentration calculated as the tetra-nucleosome concentration in the supernatant at 4 mM $MgCl_2$ divided by the average of that at 0 mM $MgCl_2$ (n=3). Error bars represent standard deviations of three independent experiments. *$p<0.05$, **$p<0.01$.

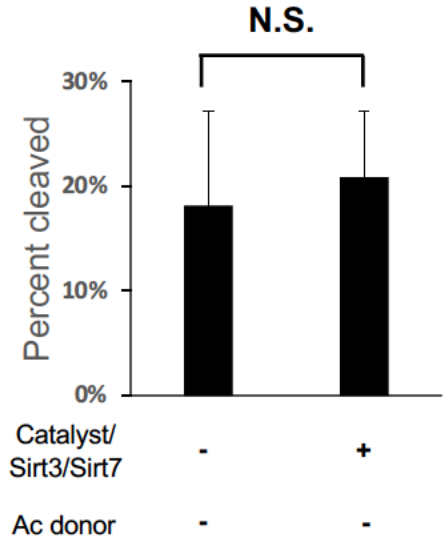

Figure S4. Control experiment in BER assay. Percentage of dU cleavage by UDG/APE1 in tetra-nucleosomes (dU-39 at N1), with or without catalyst **1**, Sirt3, and Sirt7. Each substrate was analyzed in three replicates, with data presented as the mean ± standard deviation. N.S. not significant.

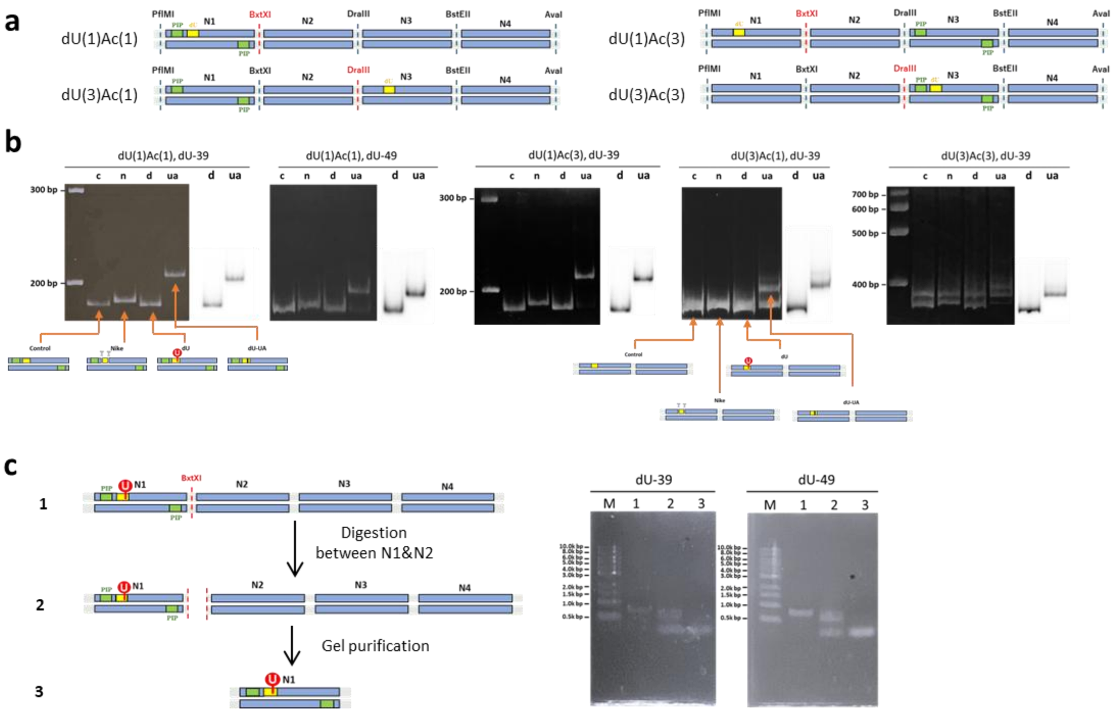

Figure S5. Preparation and validation of DNA for tetra-nucleosomes or mono-nucleosome. (a) DNA for tetra-nucleosomes used in this study, the position of the plug-and-play site (yellow) used for dU insertion, and the PIP-binding site (green) are shown. (b) Electrophoretic mobility shift assay demonstrating the insertion of dU-39 or dU-49 in DNA for tetra-nucleosomes after the plug-and-play method. The DNA was digested by the indicated restriction enzyme (red in (a)), and analyzed by 10% native PAGE (29:1 acrylamide:bisacrylamide, 1×TAE buffer, 300V). **c**: control DNA (before nicking); **n**: nicked DNA by Nb.BssSI; **d**: dU-containing DNA after the plug-and-play method; **ua**: UDG /APE1 treated dU-containing DNA. (Left) EtBr or SYBR Green staining, (Right) ^{32}P detection. (c) DNA for mono-nucleosome was excised from DNA for tetra-nucleosomes by BstXI restriction enzyme. M: 1k bp DNA ladder. DNA was visualized by SYBR Green staining.

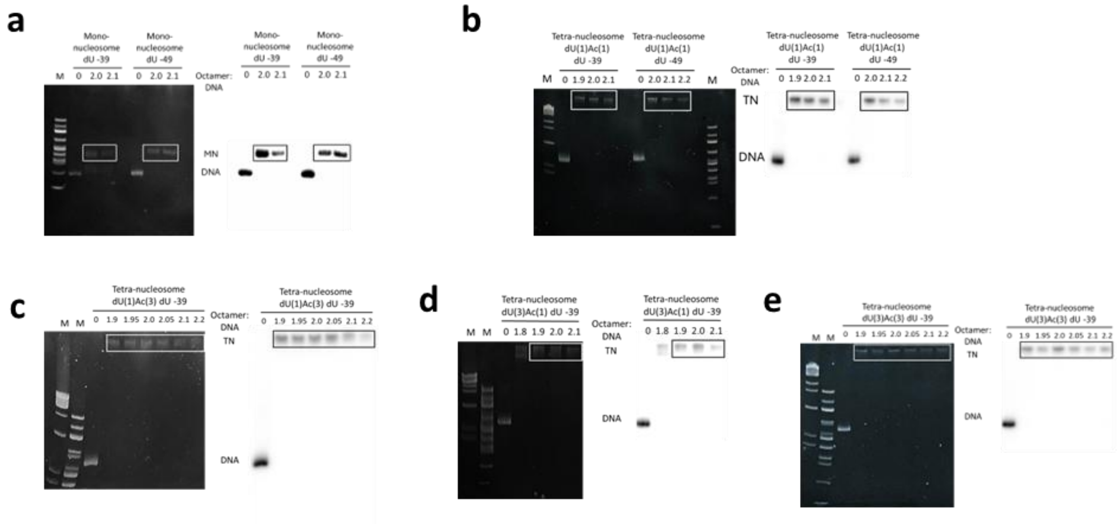

Figure S6. Analysis of mono-nucleosome and nucleosome array reconstitutions. 6% native PAGE (59:1 acrylamide:bisacrylamide, 0.2×TBE buffer, 180V) analysis of reconstituted mono-nucleosome (a) or tetra-nucleosomes (b-e). The molar ratio of histone octamer to DNA is indicated. (Left) EtBr staining, (Right) ^{32}P detection. Nucleosomes in the indicated box were collected.

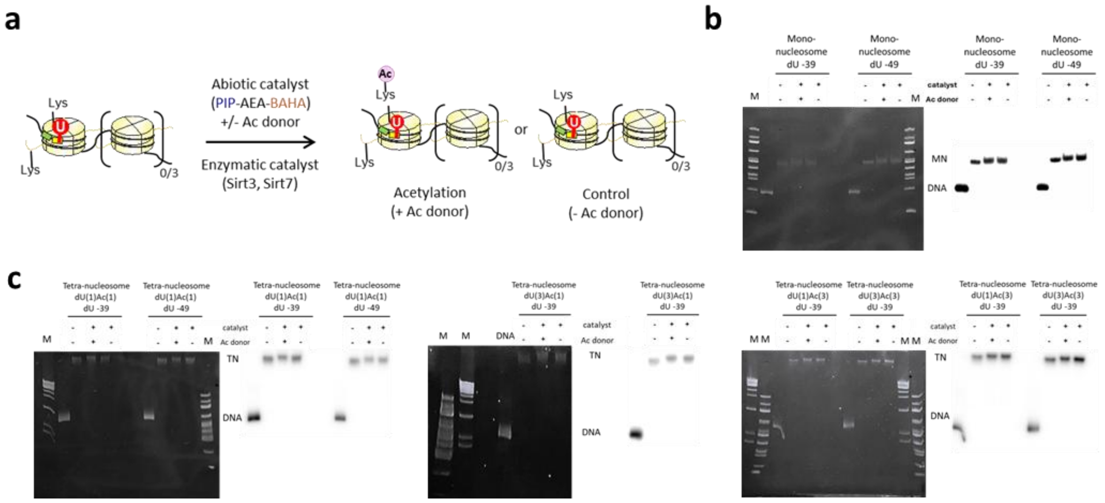

Figure S7. Analysis of acetylated or unmodified mono-nucleosome and nucleosome array. (a) Schematics of acetylated or control mono/tetra-nucleosome. (b-e) 6% native PAGE (59:1 acrylamide:bisacrylamide, 0.2×TBE buffer, 180V) analysis of acetylated or control mono/tetra-nucleosome. (Left) EtBr staining, (Right) ^{32}P detection.

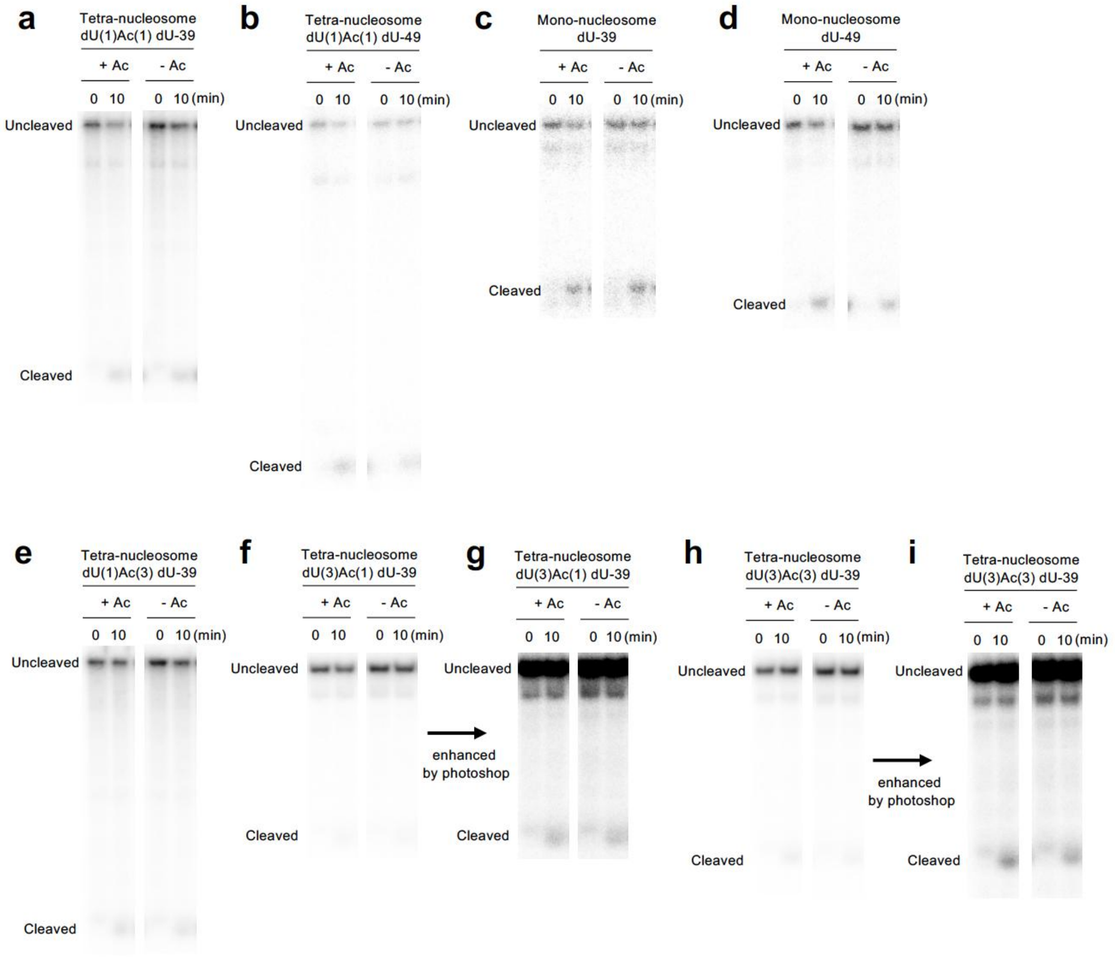

Figure S8. Raw data of BER assay. 10% denaturing PAGE gel (7 M Urea, 19:1, 1x TBE buffer, 300V) analysis was shown. (a, b) Representative raw data for Figure 3a. (c, d) Representative raw data for Figure 3b. (e, f, h) Representative raw data for Figure 4. More than three replicates were performed for each substrate. (g) The enhanced image of (f). (i) The enhanced image of (h).

Table S1. Oligonucleotides used in this study.

Name	601 unit	Sequence
N5_FWD.HindIII/PflMI	N5 (pUC-12-601)	5' -GCAATGAAGCTTCCATTGCCGCCAACTAATGG
N5_REV.XhoI/BstXI	N5 (pUC-12-601)	5' -CAAGTTCTCGAGGGGATCCGCCAAAATCGTGG
N5_PIP-bing site mut_1_FWD	N5 (pUC-12-601)	5' -TAACCGCCAAGGGGAGCCGTCCCTAGTCTCCAGGCACG
N5_PIP-bing site mut_1_REV	N5 (pUC-12-601)	5' -TCCCCTTGGCGGTTAGGACGCGGGGGACAGCGC
N5_PIP-bing site mut_2_FWD	N5 (pUC-12-601)	5' -AGAGCTGCACAGCCTGTTCCATTGCCGCCACGA
N5_PIP-bing site mut_2_REV	N5 (pUC-12-601)	5' -AGGCTGTGCAGCTCTGACACGTGCCTGGAGACT
N5_Plug-and-play site dU-39 mut_FWD	N5 (pUC-12-601)	5' -GTGCCTTGGTCCTCGTGAGCTCTAGCACCGCTTAAACGC
N5_Plug-and-play site dU-39 mut_REV	N5 (pUC-12-601)	5' -CGAGGACCAAGGCACGAGCCTCGGCACCGGGATT
N5_Plug-and-play site dU-49 mut_FWD	N5 (pUC-12-601)	5' -GTGCCCTCAATTGCTCGTGGACAGCTCTAGCACCGC
N5_Plug-and-play site dU-49 mut_REV	N5 (pUC-12-601)	5' -GAGCAATTGAGGGCACGAGGCACCGGGATTCTCCAG
N1_Ac(1)_FWD.PflMI	N1	5'-TTGCCGCCAACTAATGGCCGGATCCCCATCAGCAGTAAT GGGCCGAGGCCGCTCAATTG
N1_dU(1)Ac(1)_dU-39_FWD.PflMI	N1	5' -ATTGCCGCCAACTAATGGCCGGATCCCCATCAGCAGTAA TGGGCCGAGGCTCGTGCCTT
N1_Ac(1)_REV.BstXI	N1	5' -GATCCGCCAAAATCGTGGCGGCAATGGAAATCGCCAGTA ATGGCTGACACGTGCCTGG
N1_dU(1)Ac(1)_dU-49_FWD.PflMI	N1	5' -TTGCCGCCAACTAATGGCCGGATCCCCTGGAGAATCCCG GTGCCTCGTGCCCTCAATT
N2_FWD.BstXI	N2	5' -TTGCCGCCACGATTTTGGCGGATCCCCTGGAGAATCCC
N2_REV.DraIII	N2	5' -CCGTTGCACGTAGTGCGGCAATGGAACAGGCTGTGC
N3_FWD.DraIII	N3	5' -TTGCCGCACTACGTGATCCGGATCCCCTGGAGAAT
N3_REV.BstEII	N3	5' -GCCAAAGGTTACCCGGCAATGGAACAGGCTGTGC
N4_FWD.BstEII	N4	5' -TTGCCGGGTAACCGCATCCGGATCCCCTGGAGA
N4_REV.AvaI	N4	5' -GCCAAAACCCGAGCGGCAATGGAACAGGCTGTGC
N1_Mono-DNA_FWD	N1	5' -ATGGCCGGATCCCCATCAG
N1_Mono-DNA_REV	N1	5' -AATCGTGGCGGCAATGGAAATC
N3_site change_FWD.DraIII	N3	5' -AAAGGCACTACGTGATCCGGATCCCCATCAGC
N3_site change_REV.BstEII	N3	5' -AAAGGGGTTACCCGGCAATGGAAATCGCC
N1_site change_FWD.PflMI	N1	5' -AAAGGCCAACTAATGGCCGGATCCCCTGGAGAATC
N1_site change_REV.BstXI	N1	5' -AAAGGCCAAAATCGTGGCGGCAATGGAACAGGCTG
insert ssDNA dU-39		5' -TCGTGCCTTGG[U]CC
insert ssDNA dU-49		5' -TCGTGC[U]CTCAATTGC
Capture DNA dU-39		5' -GGACCAAGGCACGA
Capture DNA dU-49		5' -GCAATTGAGGGCACGA

Table S2. Plasmids used in this study.

Plasmid	Generation
pSAK880	pcDNA5/TO-N5 601
pSAK881	pcDNA5/TO-N5 601-PIP-binding site 1
pSAK882	pcDNA5/TO-N5 601-PIP-binding site 2
pSAK883	pcDNA5/TO-N5 601-PIP-binding site 2-Plug-and-Play (dU-39)
pSAK884	pUC19-DNA for the tetra-nucleosome dU(1)Ac(1) dU-39
pSAK885	pcDNA5/TO-N5 601-PIP-binding site 2-Plug-and-Play (dU-49)
pSAK886	pUC19-DNA for the tetra-nucleosome dU(1)Ac(1) dU-49
pSAK887	pUC19-DNA for the tetra-nucleosome dU(3)Ac(1) dU-39
pSAK889	pUC19-DNA for the tetra-nucleosome dU(3)Ac(3) dU-39
pSAK891	pUC19-DNA for the tetra-nucleosome dU(1)Ac(3) dU-39

Table S3. Raw data for Figures 3, 4, and S4.

a. Raw data of Figure 3.

dU(1)Ac(1)	Mono-nucleosome dU-39		Mono-nucleosome dU-49		Tetra-nucleosome dU-39		Tetra-nucleosome dU-49	
	Control	Acetylation	Control	Acetylation	Control	Acetylation	Control	Acetylation
1	40.0%	43.2%	20.1%	42.3%	16.6%	23.3%	34.5%	39.8%
2	42.1%	44.1%	24.1%	31.8%	12.9%	20.0%	38.4%	52.9%
3	40.3%	39.2%	22.4%	33.3%	17.8%	24.3%	25.6%	42.8%
4	36.9%	40.6%	21.6%	33.7%	—	25.0%	32.1%	55.7%
average	39.8%	41.8%	22.0%	35.3%	15.8%	23.1%	32.6%	47.8%
stdev	2.16%	2.28%	1.67%	4.77%	2.51%	2.22%	5.37%	7.67%

b. Raw data of Figure 4.

Tetra-nucleosome	dU(1)Ac(1)		dU(1)Ac(3)		dU(3)Ac(1)		dU(3)Ac(3)	
	Control	Acetylation	Control	Acetylation	Control	Acetylation	Control	Acetylation
1	11.1%	13.5%	11.3%	14.3%	0.08%	0.42%	0.42%	2.62%
2	18.4%	26.2%	11.6%	12.2%	0.60%	1.27%	0.37%	3.89%
3	15.6%	20.3%	10.9%	8.97%	0.08%	1.43%	0.44%	2.18%
4	12.1%	22.8%	20.9%	18.3%	1.23%	1.53%	0.45%	3.77%
5	16.6%	23.3%	25.2%	16.2%	1.10%	1.89%	0.93%	1.77%
6	12.9%	20.0%	18.1%	17.1%	0.91%	—	0.74%	2.53%
7	17.8%	24.3%	—	—	—	—	—	—
8	—	25.0%	—	—	—	—	—	—
average	14.9%	21.9%	16.3%	14.5%	0.67%	1.31%	0.56%	2.79%
stdev	2.87%	4.02%	6.01%	3.46%	0.50%	0.54%	0.22%	0.86%

c. Raw data of Figure S4.

dU(1)Ac(1) Tetra-nucleosome		
	without catalyst and sirtuins	with catalyst and sirtuins
1	27.9%	28.1%
2	16.5%	18.5%
3	10.2%	16.1%
average	18.2%	20.9%
stdev	9.00%	6.32%

References

1. Adamson, C.; Kajino, H.; Kawashima, S.A.; Yamatsugu, K.; Kanai, M. Live-Cell Protein Modification by Boronate-Assisted Hydroxamic Acid Catalysis. *J. Am. Chem. Soc.* **2021**, *143*, 14976–14980.
2. Banerjee, D. R.; Deckard, C. E.; Elinski, M. B.; Buzbee, M. L.; Wang, W. W.; Batteas, J. D.; Sczepanski, J. T. Plug-and-Play Approach for Preparing Chromatin Containing Site-Specific DNA Modifications: The Influence of Chromatin Structure on Base Excision Repair. *J. Am. Chem. Soc.* **2018**, *140*, 8260-8267.
3. Wang, W. W.; Zeng, Y.; Wu, B.; Deiters, A.; Liu, W. R. A Chemical Biology Approach to Reveal Sirt6-targeted Histone H3 Sites in Nucleosomes. *ACS Chem. Biol.* **2016**, *11*, 1973-1981.
4. Sczepanski, J. T.; Wong, R. S.; McKnight, J. N.; Bowman, G. D.; Greenberg, M. M. Rapid DNA-protein Cross-linking and Strand Scission by an Abasic Site in a Nucleosome Core Particle. *Pro. Natl. Acad. Sci. USA* **2010**, *107*, 22475-22480.
5. Muthurajan, U.; Mattiroli, F.; Bergeron, S.; Zhou, K.; Gu, Y.; Chakravarthy, S.; Dyer, P.; Irving, T.; Luger, K. In Vitro Chromatin Assembly: Strategies and Quality Control. *Enz. Epigen. Part A* **2016**, *573*, 3-41.
6. Tanabe, K.; Liu, J.; Kato, D.; Kurumizaka, H.; Yamatsugu, K.; Kanai, M.; Kawashima, S.A. LC–MS/MS-based quantitative study of the acyl group- and site-selectivity of human sirtuins to acylated nucleosomes. *Sci. Rep.* **2018**, *8*, 2656.
7. Garcia, B. A.; Mollah, S.; Ueberheide, B. M.; Busby, S. A.; Muratore, T. L.; Shabanowitz, J.; Hunt, D. F. Chemical Derivatization of Histones for Facilitated Analysis by Mass Spectrometry. *Nat. Protocols* **2007**, *2*, 933-938.
8. Drogaris, P.; Wurtele, H.; Masumoto, H.; Verreault, A.; Thibault, P. Comprehensive Profiling of Histone Modifications Using a Label-free Approach and Its Applications in Determining Structure-function Relationships. *Anal. Chem.* **2008**, *80*, 6698-6707.
9. Shevchenko, A.; Wilm, M.; Vorm, O.; Mann, M. Mass Spectrometric Sequencing of Proteins from Silver Stained Polyacrylamide Gels. *Anal. Chem.* **1996**, *68*, 850-858.
